# *Acinetobacter baumannii* lipooligosaccharide core region promotes CD14-dependent TLR4 endocytosis and enhances pathogenicity through interferon-β production

**DOI:** 10.1371/journal.ppat.1014364

**Published:** 2026-07-14

**Authors:** Yi-Tzu Lee, Te-Li Chen, Shu-Chen Kuo, Yu-Han Liu, Meng-Chang Lee, Ming-Fen Chuang, Mei-Chei Tan, I-Ming Lee, Ya-Sung Yang, Yung-Chih Wang

**Affiliations:** 1 Department of Emergency Medicine, Taipei Veterans General Hospital, Taipei, Taiwan; 2 Faculty of Medicine, School of Medicine, National Yang Ming Chiao Tung University, Taipei, Taiwan; 3 Graduate Institute of Life Sciences, College of Biomedical Sciences, National Defense Medical University, Taipei, Taiwan; 4 National Institute of Infectious Diseases and Vaccinology, National Health Research Institutes, Miaoli County, Taiwan; 5 Division of Infectious Diseases and Tropical Medicine, Department of Internal Medicine, Tri-Service General Hospital, National Defense Medical University, Taipei, Taiwan; 6 School of Public Health, National Defense Medical University, Taipei, Taiwan; 7 Department of Marine Biotechnology and Resources, National Sun Yat-sen University, Kaohsiung, Taiwan; 8 Institute of Biological Chemistry, Academia Sinica, Taipei, Taiwan; Washington University in Saint Louis School of Medicine, UNITED STATES OF AMERICA

## Abstract

Bacterial strains harboring lipooligosaccharides (LOS) with a core region are more pathogenic than those without it; however, the underlying mechanism remains to be fully elucidated. Lipid A has been believed to be the primary moiety of LOS that regulates Toll-like receptor 4 (TLR4) signaling, with the core region having minimal impact. In this study, we aimed to elucidate the influence of the LOS core region of *Acinetobacter baumannii* on immune response and pathogenesis. We conducted a series of step-by-step experiments and deciphered the association between the core region of the LOS and the immune response. The influence of this core region on the mechanism of TLR4 signaling pathways and further pathogenesis has also been deciphered. One *A. baumannii* wild strain with an intact LOS core region, its LOS core region gene (*lpsB*)-deficient strain, and the *lpsB* gene-complemented strain were used in this study. Membrane vesicles released from the strains were collected, quantified, normalized to the particle number, and used in subsequent experiments. Cytokine and chemokine gene expression, protein production, and RNA sequencing analyses were measured. A step-by-step approach was used to explore the underlying mechanism. The effect of the LOS core region on pathogenesis was determined using mouse experiments. Specifically, the LOS core promotes CD14-associated TLR4 endocytosis and boosting the expression of TRIF-associated genes, including *Infb1*. This enhancement in interferon-β production correlates with increased pathogenicity in an animal model. These findings highlight the significance of the LOS core region in modulating immune responses through TRIF signaling, challenging previous assumptions regarding the role of this core region in LOS-mediated pathogenesis.

## Author summary

In this study, following a series of experiments, we determined that the core region of lipooligosaccharides (LOS) enhanced CD14-dependent toll-like receptor 4 (TLR4) endocytosis. It further enhanced interferon-β (IFN-β) production through the TLR4-TRIF signaling pathway and contributed to higher pathogenicity. Initially, we found that an *A. baumannii* strain harboring the core region of LOS displayed higher virulence than a strain without this core region. We further showed that the membrane vesicles (MVs) released from *A. baumannii* with intact core stimulated greater IFN-β production and higher *Infb1* gene expression than those released from *A. baumannii* without intact core. The core region of LOS upregulated the expression of IFN-β-associated genes, as revealed from the RNA sequencing analysis. We further confirmed that IFN-β production was stimulated through the CD14-TLR4-TRIF signaling pathway. In animal experiments, IFNAR1-deficient mice (Ifnar1-/-) exhibited a higher survival rate than did the wild-type mice after challenge with MVs containing an intact LOS core. Treatment with anti-IFN-β antibodies, but not isotype control antibodies, significantly improved the survival of mice exposed to MVs released from *A. baumannii*. We believe that our study makes a significant contribution because it demonstrates that the core region of LOS promotes CD14-associated TLR4 endocytosis, thereby increasing the expression of TRIF-associated genes, including *Infb1*, and further enhances the pathogenesis of *A. baumannii*.

## Introduction

*Acinetobacter baumannii* is a leading pathogen of nosocomial infections [1,2], and is notorious for its rapid evolution of resistance to numerous antimicrobial agents [3,4]. Despite extensive studies on resistance mechanisms, the pathogenesis of *A. baumannii* infection has not been sufficiently studied [3,5]. In gram-negative bacteria, lipopolysaccharide (LPS) is a major virulence factor comprising lipid A and a core oligosaccharide that includes an outer core, inner core, and a polysaccharide repeat (O antigen) [6,7]. *A. baumannii* possesses lipooligosaccharide (LOS), a molecule similar to LPS, but without the O antigen [8]. LPS/LOS appear as micelles or actively secreted membrane vesicles (MVs) when released into physiological fluids [7,9]. LPS/LOS monomers are extracted from MVs by LPS/LOS binding protein (LBP) and transferred to CD14 [10,11], the Toll-like receptor 4/myeloid differentiation factor 2 (TLR4/MD-2) complex, followed by dimerization of the TLR4/MD-2 complex and activation of the MyD88 and/or TRIF signaling pathway [10,12].

Researchers believe that TLR4 signaling in LPS/LOS depends on the lipid A moiety [13,14] and that the core region does not significantly modulate this signaling [10]. Deletion of the *lpsB* gene results in loss of the LOS core region, leaving only 3-deoxy-D-manno-oct-2-ulsonic acid 9 (KDO) and lipid A on LOS (designated as Re-LOS hereafter) [7]. Compared with LOS, Re-LOS results in lower serum resistance *in* *vitro*, survival fitness *in vivo* [15], and virulence in a mouse pneumonia model [16].

In this study, we demonstrated that the core region of *A. baumannii* LOS contributes substantially to immune modulation by selectively enhancing one of the TLR4 signaling pathways. Mouse pneumonia and an intraperitoneal sepsis model with C57BL/6 mice were used in animal experiments to elucidate the pathogenesis of *A. baumannii* LOS. The LOS core region significantly promoted CD14-associated TLR4 endocytosis and consequently upregulated the expression of IFN-β, which correlated with increased pathogenicity in the animal model.

## Results

### Bacterial strains

The plasmid used for generating *lpsB* gene knockout *A. baumannii* mutants is shown in S1a Fig. S1b Fig shows a schematic representation of *A. baumannii* strains harboring different LOS lengths. S1c Fig presented PCR mapping results confirming the successful double-crossover deletion of the *lpsB* gene in strain Ab908*ΔlpsB*. Silver stain of the purified LOS confirmed that Ab908 and Ab908*ΔlpsB*::*lpsB* produce LOS with core, but not Ab908*ΔlpsB* (S1d Fig). The three strains exhibited comparable growth *in vitro* (S1e Fig).

### *0.1 A. baumannii* with intact LOS demonstrated higher pathogenicity than those lacking the core region

**Fig 1:**
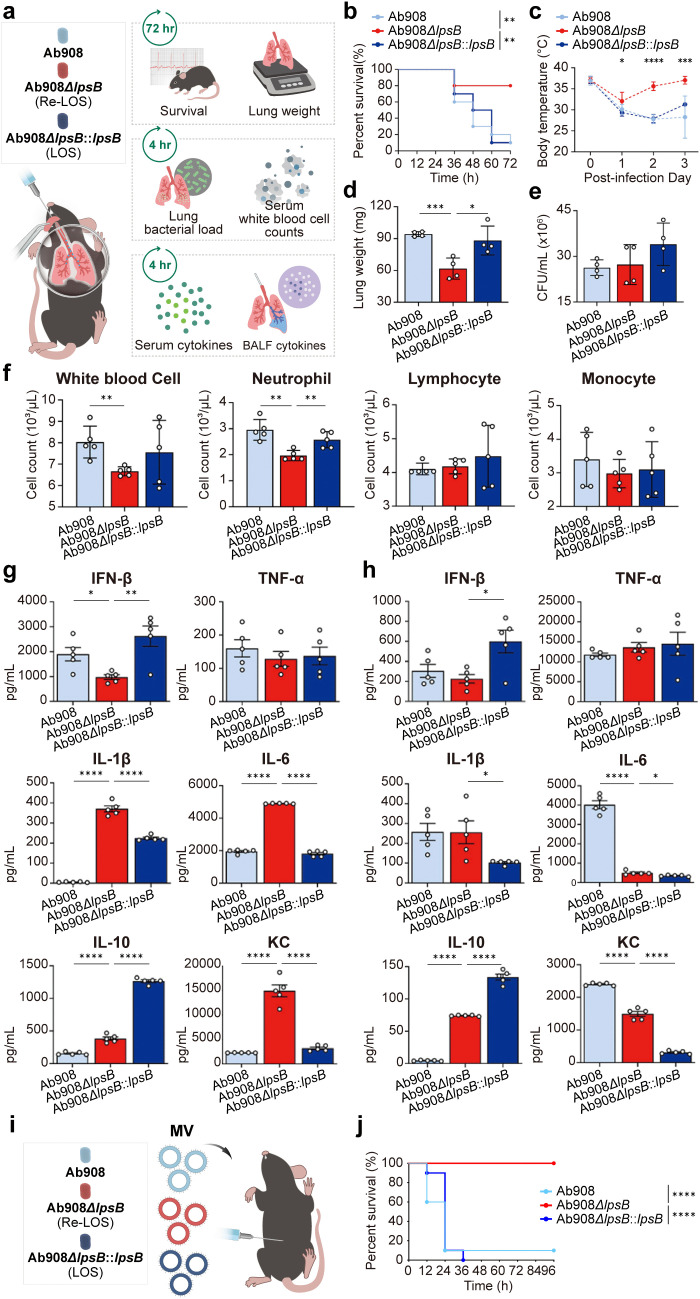
*Acinetobacter baumannii* strains with intact lipooligosaccharide (LOS) (Ab908 and Ab908*ΔlpsB::lpsB*) demonstrated higher virulence than those lacking the core region of LOS (Re-LOS, Ab908*ΔlpsB*). **(a)-(h)** Female C57BL/6 mice were intratracheally injected with 5 × 10^8^ colony-forming unit (CFU) of live *A. baumannii*. **(a)** Schematic illustration of the experimental design. **(b)** Mouse survival for 72 h (n = 10 in each group). **(c)** Body temperature was measured daily for 3 days (n = 10 in each group at the beginning of the experiments). **(d)** Weights of lungs harvested 3 days post-infection (n = 4 in each group). **(e)** Bacterial count in lung tissues 4 h (n = 4 in each group). **(f)** White blood cell and differential cell counts in serum collected 4 h post-infection. **(g)** Cytokine and chemokine levels in serum collected 4 h post-infection (n = 5 in each group). **(h)** Cytokine and chemokine levels in bronchoalveolar lavage fluid collected 4 h post-infection (n = 5 in each group). **(i)-(j)** Data of female C57BL/6 mice injected intraperitoneally with *A. baumannii* membrane vesicles (MVs)(~8.6 × 10^11^ particles in 100 μl; n = 10 in each group). **(i)** Schematic of the experimental design. **(j**) Mouse survival observed for 96 **h.** Statistical analysis: **(c)-(h)** unpaired two-tailed Student’s t-test: *P < 0.05; **P < 0.01; ***P < 0.001; ****P < 0.0001. In Fig **(c)**, the comparison was made between Ab908 and Ab908*ΔlpsB.* (**b)(j)** Kaplan–Meier analysis, log–rank test: **P < 0.01; ****P < 0.0001.

A schematic diagram of the experimental design is shown in Fig 1a. Mice infected with strains harboring intact LOS displayed higher mortality rates (Fig 1b), lower body temperatures (Fig 1c), and greater lung weights (Fig 1d) than those that did not.

The bacterial load of the strain lacking the core region (Re-LOS) did not significantly differ from that of the strain with intact LOS at 4 h post-infection (Fig 1e). Serum white blood cell and neutrophil counts were significantly higher in mice exposed to the strain harboring an intact LOS 4 h post-infection (Fig 1f). The results showed that factors other than the bacterial burden may be responsible for the changes during the early phase of infection (4 h post-infection). In the serum and bronchoalveolar lavage fluid (BALF), the *A. baumannii* strain with intact LOS induced higher interferon beta (IFN)-β but not tumor necrosis factor (TNF)-α secretion (Fig 1g and 1h). Other cytokines/chemokines did not show consistent results in the serum and BALF (Fig 1g and 1h).

We used MVs in subsequent experiments to mitigate potential confounding factors associated with live bacteria that could influence pathogenicity (Fig 1i). The MVs released from the strains harboring intact LOS resulted in significantly higher fatality rates than those released from the strains with Re-LOS (Fig 1j). These results revealed that the LOS core region was associated with the enhanced pathogenicity of *A. baumannii* strains.

### MVs containing intact LOS induced higher levels of IFN-β release and greater responsiveness of IFN-β-related genes in macrophages than those containing Re-LOS

We found that two cytokines, IFN-β and TNF-α, which are induced upon recognition of LOS by TLR4 [7,12], exhibited distinct responses in mice stimulated with *A. baumannii* harboring intact LOS versus Re-LOS. Therefore, we aimed to delineate whether strains carrying intact LOS or Re-LOS could elicit differential levels of IFN-β and TNF-α in immune cells. Macrophages were selected based on their important role as frontline innate immune cells during bacterial invasion and exposure to virulence factors.

**Fig 2:**
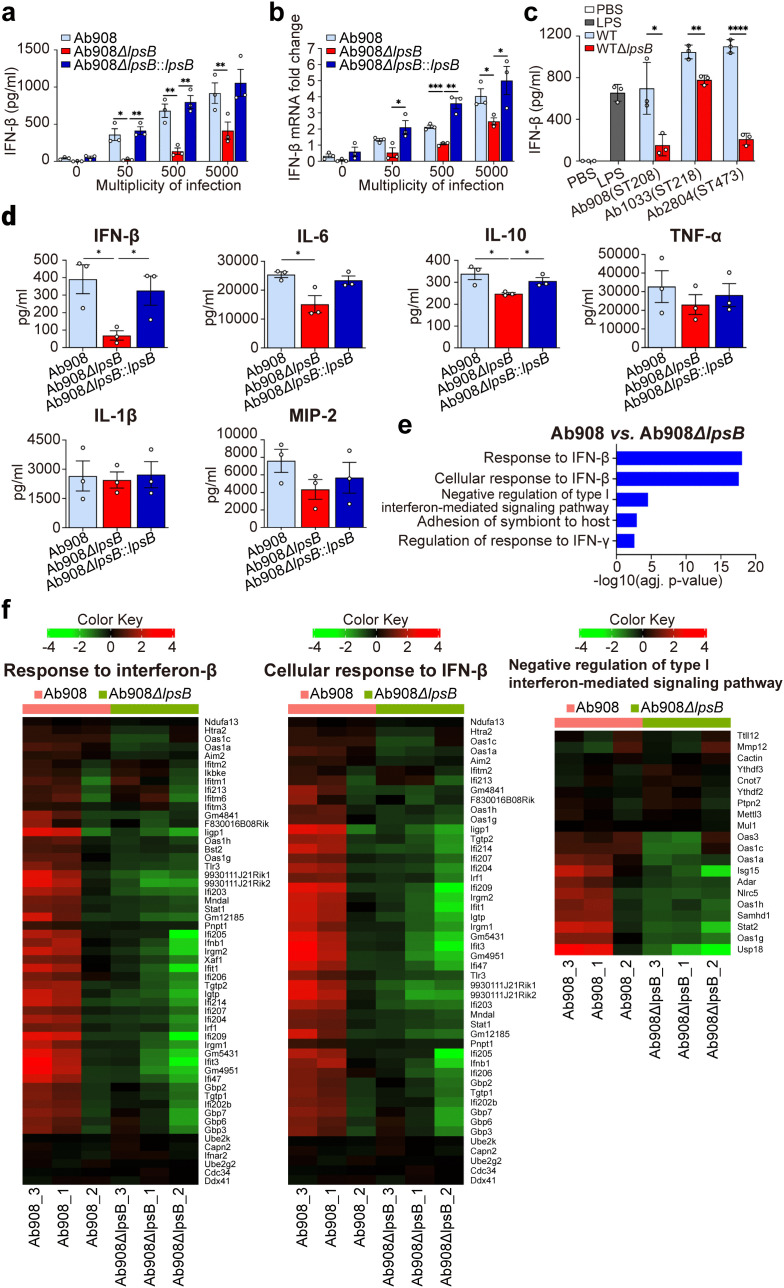
Membrane vesicles containing intact lipooligosaccharide (LOS) induced increased IFN-β secretion and higher *Infb1* gene expression and its signaling pathway than those lacking the core region of LOS (Re-LOS). **(a)** Interferon (IFN)-β secretion and **(b)** *Infb1* gene expression in murine macrophage J774a.1 cells stimulated with membrane vesicles (MVs) at the indicated concentrations for 4 **h. (c)** IFN-β secretion 6 h after stimulation with MVs (multiplicity of infection [MOI]=500) from *lps*B knockout (Δ*lpsB*) strains and their parent wild-type (WT) *A. baumannii* strains belonging to different sequence types. **(d)** Cytokine levels in THP1-Dual-derived macrophages 24 h after stimulation with MVs from different strains (MOI = 500). The means and standard error of the means from three biological replicates are shown, with significance being determined using an unpaired two-tailed Student’s t-test. *P < 0.05; **P < 0.01; ***P < 0.001; ****P < 0.0001. **(e)** Top 5 biological processes identified using gene set enrichment analysis comparing responses to MVs from Ab908 (with intact LOS) and Ab908*ΔlpsB* (with Re-LOS) **(f)** Heatmaps illustrating the expression of genes related to “response to IFN-β”, “cellular response to IFN-β”, and “negative regulation of type I interferon-mediated signaling pathway” in J774a.1 cell in response to the indicated stimuli. The scale numbers of the color keys represent log₂(FC). All data were retrieved from three biological replicates.

The mouse macrophages stimulated with the MVs containing intact LOS secreted significantly more IFN-β than those stimulated with MVs containing Re-LOS, in a dose-dependent manner (Fig 2a). Moreover, the MVs with intact LOS elicited higher levels of *Infb1* expression than those with Re-LOS did, in a dose-dependent manner (Fig 2b). The enhanced induction of IFN-β was consistent across the different *A. baumannii* strains belonging to various sequence types (Fig 2c). We showed that MVs containing intact LOS also induced significantly more IFN-β, IL-6, and IL-10 production in human macrophages (Fig 2d). In contrast, there was no significant difference in the production of TNF-α, IL-1β, and MIP-2 production among the groups (Fig 2d).

To examine the gene expressions, murine macrophage J774a.1 cells stimulated with MVs of Ab908 and Ab908*ΔlpsB* were used for RNA sequencing. RNA sequencing analysis identified 134 differentially expressed genes (DEGs) in the Ab908 MV group compared with the Ab908*ΔlpsB* MV group (with Re-LOS), of which 132 were upregulated and 2 were downregulated (S2a Fig). *Infb1* expression was significantly upregulated in the Ab908 MV group (with an intact LOS; S2b Fig). Gene Set Enrichment Analysis (GSEA) revealed that the top five upregulated biological processes were “response to IFN-β,” “cellular response to IFN-β,” “negative regulation of type I interferon-mediated signaling pathway,” “adhesion of symbiont to host,” and “regulation of response to IFN-γ” as shown in Fig 2e. Heatmap analysis (Fig 2f) depicted that the expression of *Irf1* and *Infb1* was significantly upregulated in “response to IFN-β” and “cellular response to IFN-β,” whereas that of *Stat2* was upregulated in “negative regulation of type I interferon-mediated signaling pathway.” These results showed that the MVs containing intact LOS induced higher levels of IFN-β release and greater responsiveness of IFN-β-related genes in macrophages than those containing Re-LOS.

### Core region of LOS enhanced IFN-β production through the TLR4-TRIF signaling pathway

**Fig 3:**
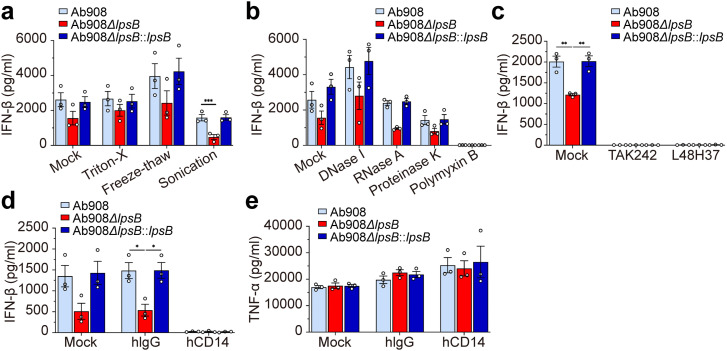
The core region of lipooligosaccharide (LOS) is responsible for enhanced IFN-β production. **(a)** Interferon (IFN)-β secretion in J774a.1 macrophages pre-treated with Triton X-100, subjected to freeze–thaw cycles, or sonicated (2 cycles of 25 s each with 5 s pause at 30% power on ice) before being stimulated with Ab908 membrane vesicles (MVs). **(b)** IFN-β secretion in J774a.1 macrophages pre-treated with DNase I, RNase A, proteinase K, or polymyxin B for 30 min before being stimulated with MVs. **(c)** IFN-β secretion in THP1-Dual-derived macrophages pre-treated with TAK242 (Toll-like receptor 4 [TLR4] inhibitor) for 1 h and L48H37 (Myeloid differentiation factor-2 [MD-2] inhibitor) for 0.5 h before being stimulated with MVs. **(d)** IFN-β and **(e)** TNF-α secretion in THP1-Dual-derived macrophages pre-treated with anti-human CD14 antibody (hCD14) or anti-human IgG (hIgG, control) for 1 h before being stimulated with MVs (multiplicity of infection [MOI] = 500) from different bacterial strains. Cytokines were measured using an enzyme-linked immunosorbent assay 4 h after stimulation. The concentrations used were as follows: Triton X-100 (0.1% vol/vol); DNase I, 1 U/μL; RNase A, 200 μg/mL; proteinase K, 100 μg/mL; polymyxin B, 3 μM; TAK242, 1 μM; L48H37, 10 μM; hIgG, 10 μg/mL; hCD14, 10 μg/mL. Statistical significance was determined using an unpaired two-tailed Student’s t-test. *P < 0.05; **P < 0.01, ***P < 0.001.

LPS located within complex particles, such as MVs and intact gram-negative bacteria, may selectively promote TRIF-dependent TLR4 signaling [17,18]. *lpsB* deletion results in the loss of the LOS core, potentially altering the structure of the bacterial membrane [19] and indirectly affecting the characteristics and content of the MVs released from the strain with Re-LOS. For example, the MVs of the strain carrying Re-LOS were slightly larger than those of the strain carrying intact LOS (S1 Table). These changes could be responsible for the lower production of IFN-β in the macrophages stimulated with the MVs containing Re-LOS. However, compared with the MVs containing Re-LOS, those with intact LOS still induced higher IFN-β levels even after chemical or physical disruption (Fig 3a). We further clarified that neither DNase I, RNase A, nor proteinase K treatment altered the differences in IFN-β production (Fig 3b). IFN-β was abolished by polymyxin B, indicating that LOS and Re-LOS of the MVs substantially influenced IFN-β secretion (Fig 3b). Addition of the TLR4 receptor inhibitor TAK-242 and MD-2 inhibitor L48H37 abolished MV-induced IFN-β secretion, suggesting that the IFN-β secretion induced by the MVs containing LOS and Re-LOS was associated with TLR4 and MD-2 (Fig 3c). While CD14 is required for LPS-induced TLR4 endocytosis, treatment with an anti-CD14 antibody abolished MV-induced IFN-β secretion (Fig 3d) but not MV-induced TNF-α secretion (Fig 3e). These results indicated that the LOS core region, but not the configuration or other components (DNA, RNA, proteins) of MVs, was responsible for increasing IFN-β secretion through the TLR4-MD-2 signaling pathway.

### LOS core region selectively enhances the TRIF-dependent pathway, but without affecting the MyD88-dependent pathway

**Fig 4:**
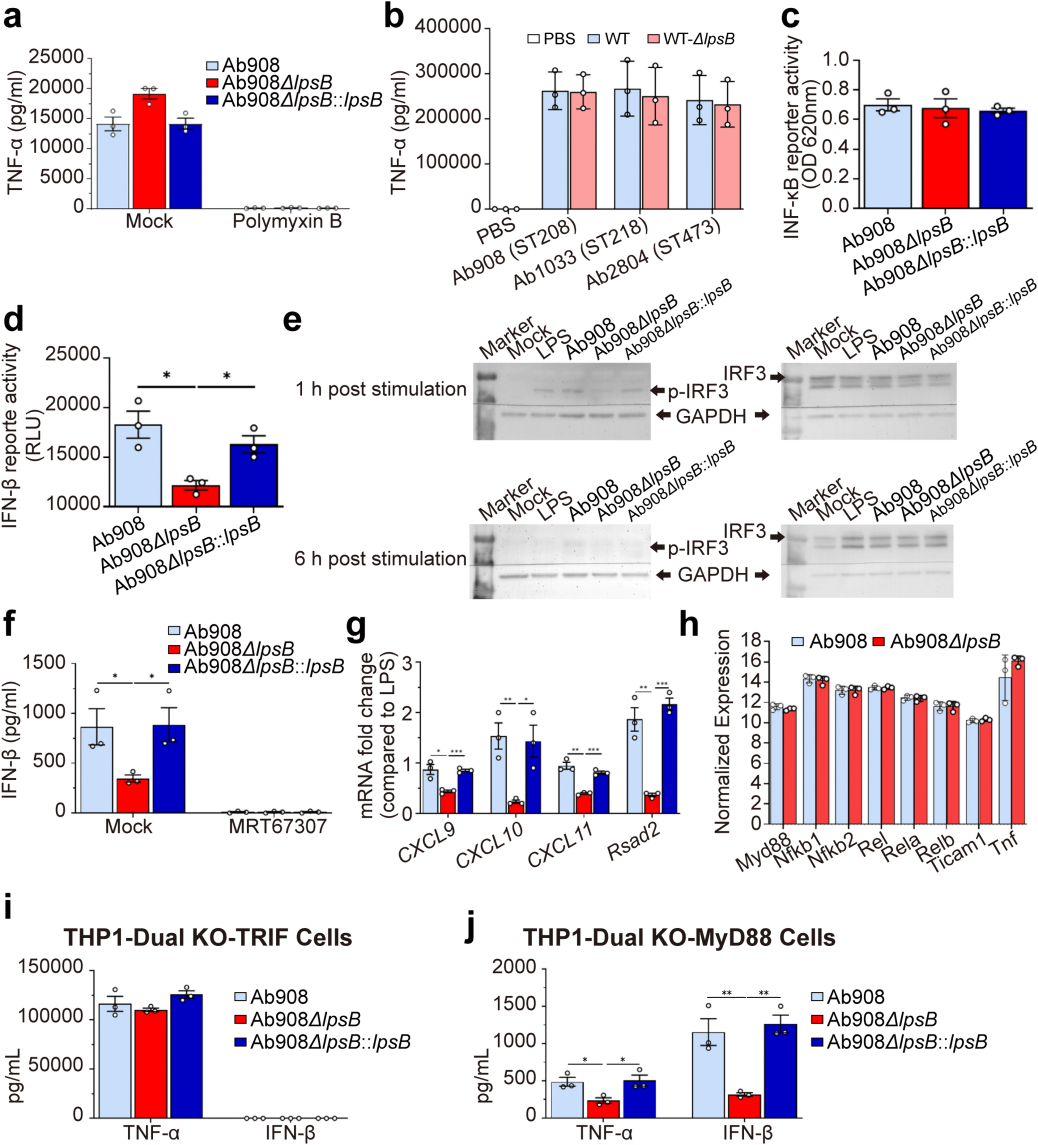
The core region of lipooligosaccharide (LOS) selectively enhances the TRIF-dependent signaling pathway but not the MyD88-dependent signaling pathway. **(a)** Tumor necrosis factor (TNF)-α production in THP1-Dual-derived macrophages pre-treated with polymyxin B (3 μM) and subsequently stimulated with membrane vesicles (MVs) from different bacterial strains (multiplicity of infection [MOI] =500). **(b)** TNF-α levels in THP1-Dual-derived macrophages 6 h after being stimulated with MVs released from the parent strains and their Re-LOS mutants belonging to different sequence types. **(c)-(d)** Activation of NF-κB and interferon regulator factor (IRF) pathways in THP1-Dual cells based on the activities of secreted embryonic alkaline phosphatase (SEAP) and luciferase, respectively. **(e)** Expression of interferon regulatory factor 3 (IRF3) and phosphorylated IRF3 (p-IRF3) in J774a.1 cells at 1 h and 6 h post-stimulation with MVs from different strains. **(f)** IFN-β production at 4 h post-stimulation with MVs (MOI = 500) in the presence or absence of IKKε/TBK1 inhibitor MRT67307 (10 μM, pre-treated for 1 **h)**. **(g)** mRNA expression in J774a.1 cell at 2 h post-stimulation with MVs. **(h)** Expression levels of MyD88-associated genes in cells stimulated with MVs. **(i)-(j)** TNF-α and IFN-β secretion in macrophages derived from **(i)** THP1-Dual KO‑TRIF cells and **(j**) THP1-Dual KO‑MyD88 cells at 6 h post-stimulation with MVs. Means and standard errors of the means from the three biological replicates are presented, with significance determined using unpaired two-tailed Student’s t-test: *P < 0.05; **P < 0.01; ***P < 0.001; ****P < 0.0001. LPS, lipopolysaccharide.

TNF-α secretion was abolished by pre-treatment with polymyxin B, indicating that the MV-induced TNF-α production was also associated with LOS (Fig 4a). The MVs with intact LOS from isolates belonging to different sequence types stimulated equivalent TNF-α secretion (Fig 4b) compared with their Re-LOS mutants. These results suggest that the LOS core selectively enhances the TRIF-dependent signaling pathway but not the MyD88-dependent signaling pathway.

MVs containing either LOS or Re-LOS equally activated the NF-κB pathway (Fig 4c); however, only MVs with LOS enhanced the activation of the interferon regulatory factor 3 (IRF3) pathway (Fig 4d). Stimulation with LOS, but not Re-LOS, resulted in the phosphorylation of IRF3 at an early time point (Fig 4e). IFN-β production was eliminated after MRT67307 addition, an IKKε/TBK1 inhibitor (Fig 4f). These findings revealed that the MVs stimulated the release of IFN-β through the IRF3 pathway.

Several TRIF-dependent cytokine genes, including *CXCL9*, *CXCL10*, *CXCL11*, and *Rsad2*, were significantly upregulated after stimulation with MVs containing intact LOS (Fig 4g). MVs containing either LOS or Re-LOS induced similar levels of MyD88-associated gene expression (Fig 4h). IFN-β production was eliminated in a cell line lacking TRIF (Fig 4i) but preserved in the ones lacking MyD88 (Fig 4j), indicating that the LOS-enhanced IFN-β production was solely dependent on the TLR4-TRIF signaling pathway.

The top five enriched transcription factor (TF)-binding motifs were Stat2, Irf2, Irf1, Prdm1, and Irf3 (S2 Table), indicating that the upregulated DEGs were regulated by these TFs and may not be associated with the MyD88-dependent signaling pathway. Collectively, we demonstrated that the LOS core region selectively enhanced the TRIF-dependent signaling pathway but not the MyD88-dependent signaling pathway.

### Core of LOS enhanced CD14-dependent TLR4 endocytosis

**Fig 5:**
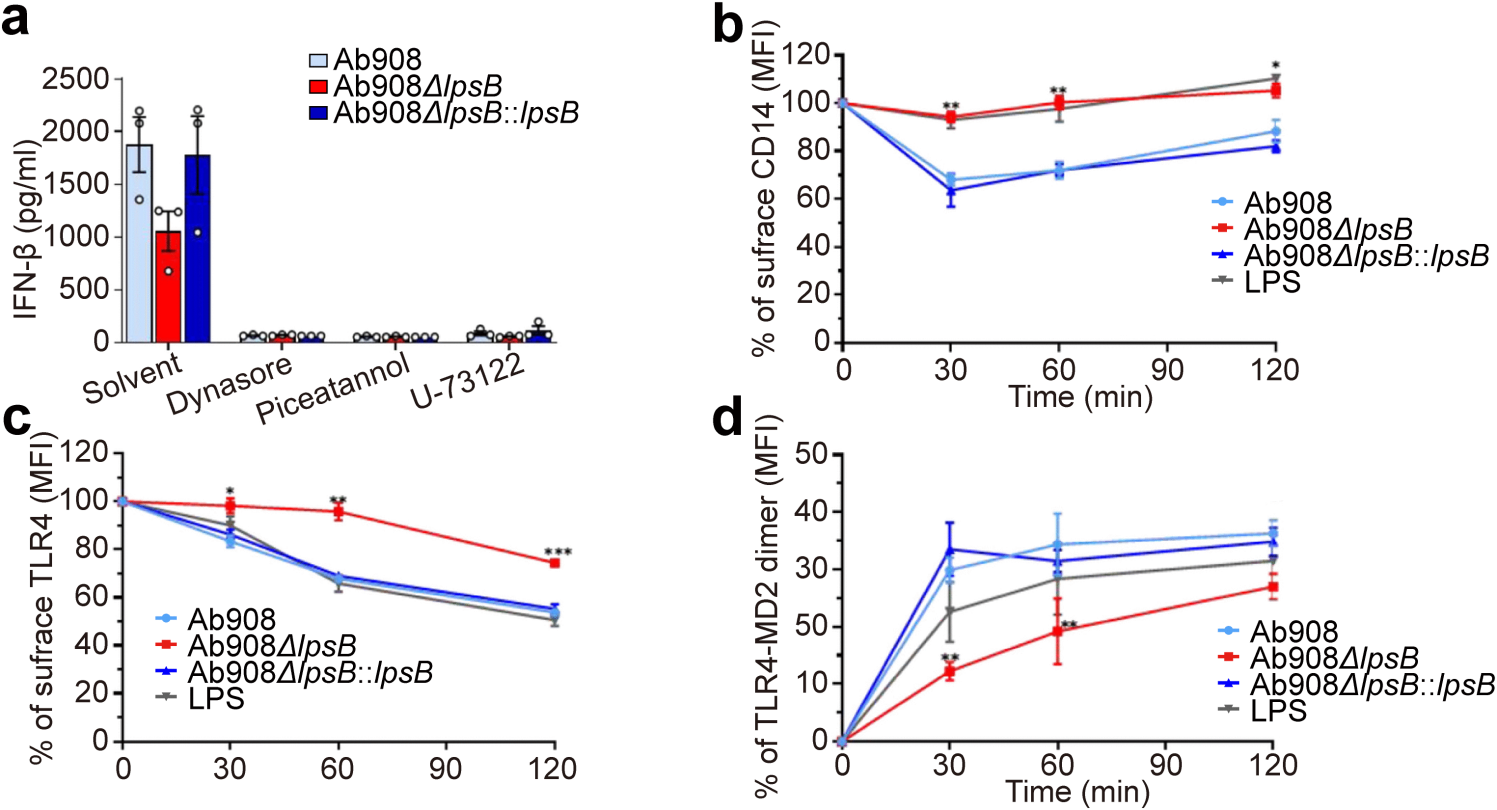
The core region of lipooligosaccharide (LOS) enhances CD14 endocytosis. **(a)** Interferon (IFN)-β measurement in THP1-Dual-derived macrophages (5 × 10^5^/well) pre-treated with dynasore (endocytosis inhibitor, 80 μM) for 1 h, piceatannol (Syk inhibitor, 75 μM), or U-73122 (PLCγ2 inhibitor, 5 μM) for 0.5 h before being stimulated with membrane vesicles (MVs) from different strains (multiplicity of infection [MOI] = 500, 4 **h)**. n = 3 biological replicates per group. **(b)-(d)** Flow cytometry results of endocytosis of **(b**) CD14, **(c**) Toll-like receptor 4 (TLR4), and **(d)** TLR4/Myeloid differentiation factor-2 (MD-2) dimerization in J774a.1 cells stimulated with MVs for the indicated times. Error bars represent mean ± standard error of the mean (SEM) from triplicate experiments. MFI, mean fluorescence intensity. The means and standard error of mean from three biological replicates are presented, with significance determined by unpaired two-tailed Student’s t-test: *P < 0.05; **P < 0.01; ***P < 0.001. LPS, lipopolysaccharide.

Dynasore (endocytosis inhibitor) obliterated IFN-β production, indicating that TLR4 endocytosis is necessary for MV-stimulated IFN-β secretion (Fig 5a). The inhibition of spleen tyrosine kinase (Syk) with piceatannol and its downstream phospholipase Cγ2 (PLCγ2) with U-73122 also eliminated the induction of IFN-β secretion by both MV stimulation groups (Fig 5a). These results indicate that LOS and Re-LOS induced IFN-β through CD14-dependent TLR4 endocytosis and TRIF-dependent signaling pathway. Unexpectedly, MVs with intact LOS enhanced the endocytosis of CD14 (Fig 5b) and TLR4 (Fig 5c), as well as TLR4/MD-2 dimerization (Fig 5d), compared with MVs with Re-LOS. These results revealed that LOS increased IFN-β production by promoting CD14-associated TLR4 endocytosis.

### IFN-β stimulated by the MVs with intact LOS is associated with increased pathogenicity

**Fig 6:**
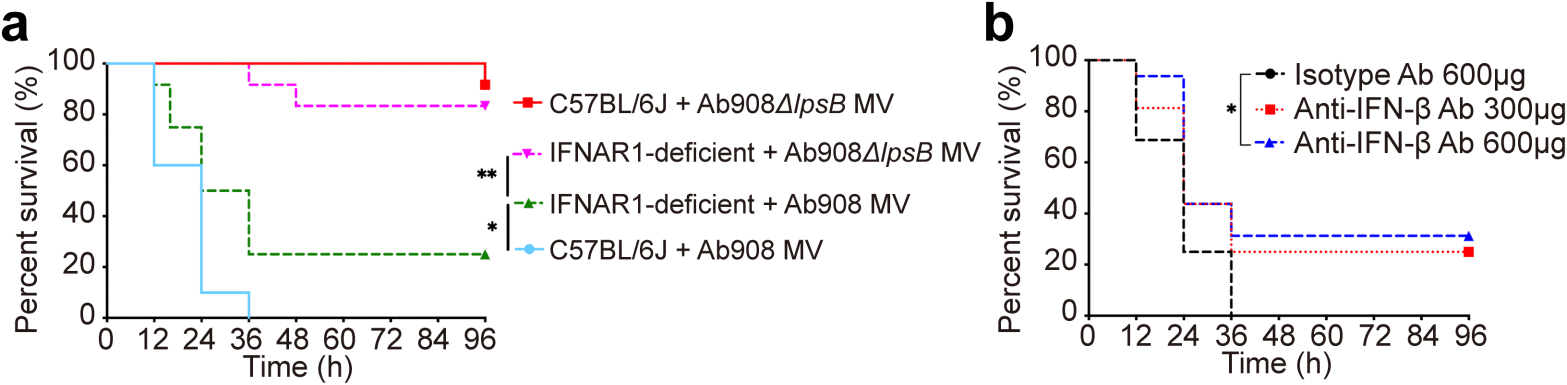
Increased IFN-β production was associated with enhanced pathogenicity in lipooligosaccharide(LOS)-treated mice. **(a)** Survival rates of female C57BL/6 and IFNAR1-deficient mice (Ifnar1-/-) that were intraperitoneally injected with *A. baumannii* MVs containing intact LOS/Re-LOS (Ab908/Ab908*ΔlpsB*) (~9.7 × 10^11^ particles in 100μL; n = 10–12 in each group). **(b)** Survival rates of female C57BL/6 mice that were intraperitoneally injected *A. baumannii* with intact LOS (Ab908) MVs (~9 × 10^11^ particles in 100μL). Anti-interferon-β antibody and isotype antibody were used 30 min after MVs injection (n = 16 in each group). Kaplan–Meier analysis was conducted. (*P < 0.05, log–rank test).

Wild-type C57BL/6 and IFNAR1-deficient mice (Ifnar1-/-), which lacked the type I IFN-α/β receptor, were intraperitoneally injected with MVs containing either intact LOS or Re-LOS. The results showed that IFNAR1-deficient mice had a higher survival rate than wild-type mice when challenged with MVs containing intact LOS (Fig 6a). However, the survival of IFNAR1-deficient mice differed significantly between the mice challenged with MVs containing intact LOS and those challenged with Re-LOS-containing MVs. Treatment with an anti-IFN-β antibody significantly reduced mortality in mice exposed to MVs with intact LOS, in a dose-dependent manner, compared with mice treated with an isotype control antibody (Fig 6b). These findings indicate that LOS-induced IFN-β production contributes to the increased pathogenicity of *A. baumannii* in mice. In addition to IFN-β, intact LOS may also contribute to increased pathogenicity through additional mechanisms.

## Discussion

**Fig 7:**
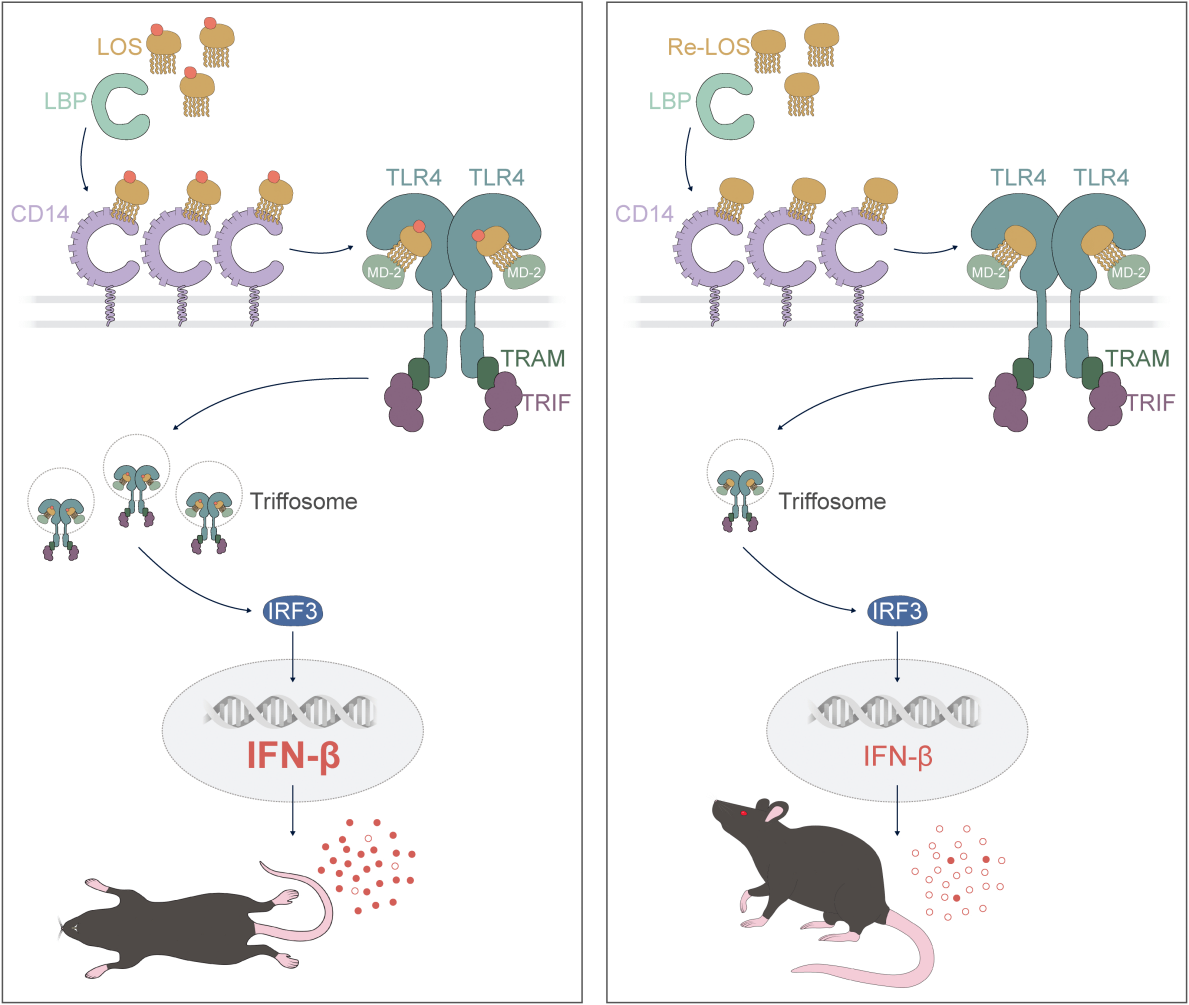
*Acinetobacter baumannii* lipooligosaccharide(LOS) core region promotes CD14-dependent TLR4 endocytosis and enhances pathogenicity through IFN-β production. Schematic illustration of the findings in this research. Created with BioArt Source (https://bioart.niaid.nih.gov/) and Wikimedia Commons (https://commons.wikimedia.org/).

Gram-negative bacteria such as *Neisseria meningitidis*, *Haemophilus influenzae*, and *A. baumannii* produce LOS, which play a critical role in evading host immune responses. The core region of LOS often mimics the host cellular components, enabling these bacteria to avoid detection [7,16]. We found an extended role for the LOS core in immune modulation, particularly in its ability to selectively enhance one of the TLR4 signaling pathways. Specifically, the core region of *A. baumannii* LOS significantly promoted CD14-associated TLR4 endocytosis and augmented IFN-β production via the TRIF-dependent signaling pathway, ultimately contributing to increased pathogenicity (Fig 7).

Our results demonstrated that certain cytokines/chemokines showed inconsistent levels in mouse serum and BALF. These discrepancies may reflect differences in the extent of local airway versus systemic inflammation [20], as well as inter-individual variability among mice. Several studies have reported that cytokine levels in BALF and serum do not always increase consistently in patients with pneumonia [21,22]. However, the two cytokines IFN-β and TNF-α, which are induced upon recognition of LOS by TLR4, exhibited distinct responses both in blood and BALF, prompting us to further investigate this phenomenon using a cell model.

Lipid A is traditionally considered the primary moiety of LPS/LOS that modulates TLR4 signaling because of its acyl and phosphate groups, which directly interact with MD-2 and TLR4 [10]. Modifications of lipid A can significantly affect TLR4 signaling [23]. For example, hypoacylated LPS reduces the activation of MyD88- and TRIF-dependent signaling pathways [24]. Recently, findings showed that the KDO moieties of the inner core interact with TLR4, enhancing the potency of lipid A in TLR4 activation [25–27].

However, the role of the saccharide components of LPS/LOS in TLR4 signaling modulation remains poorly understood. For example, the O antigen of *Salmonella* LPS delays TLR4-mediated recognition and enhances the resistance to macrophage phagocytosis [28,29]. Our results indicate that because of the LOS core, TLR4 signaling can be specifically biased toward the TRIF-dependent signaling pathway without affecting the MyD88-dependent signaling pathway. A previous study revealed that the intact LPS of *Escherichia coli* (smooth LPS [sLPS]) and its Re-LPS mutant were similarly efficient in activating the MyD88-dependent pathway. However, sLPS was slightly more efficient than Re-LPS in activating the TRIF-dependent pathway [30]. Additionally, LPS core truncation in *E. coli* has been associated with a reduced ability to invade epithelial cells [30]. *Pseudomonas aeruginosa* loses its core saccharides during chronic infection [31]. These findings reveal that the core region of LPS/LOS can modulate specific signaling pathways that contribute to pathogenicity.

Our results show that TLR4 could not differentiate between LOS and Re-LOS cells when CD14 expression was blocked. Thus, CD14 can discriminate between LOS and Re-LOS. Similarly, CD14 has been shown to distinguish between LPS with different saccharide structures [32], demonstrating that LPS with the O antigen (sLPS) is more dependent on CD14 than that without the O antigen (rLPS) for transport to TLR4, thereby initiating the MyD88-dependent signaling pathway [30,33]. Additionally, sLPS is more efficient in activating CD14-endocytosis-associated nuclear factor of activated T-cell pathways in dendritic cells [30]. These findings indicate that CD14 recognizes specific saccharide components of LPS/LOS to exert distinct immunomodulatory functions.

While IFN-β is beneficial in most acute viral infections, its role in bacterial infections can be either advantageous or harmful, depending on factors such as bacterial species, burden, duration, and location of induction [34,35]. Our results showed that enhanced IFN-β production by the core region of *A. baumannii* LOS correlated with increased pathogenicity. Using mice stimulated with *E. coli* MVs, it was previously demonstrated that genetic deletion of TRIF reduced lethality, indicating that the TRIF-dependent signaling pathway is linked to higher mortality [36].

Not all IFNAR1-deficient mice (Ifnar1-/-) survived the challenge with MVs containing intact LOS, and their survival rates were lower than those of mice challenged with MVs containing Re-LOS. These findings reveal that, in addition to IFN-β, intact LOS may contribute to increased pathogenicity through additional mechanisms. For example, an intact LOS may enhance resistance to phagocytosis or complement-mediated disruption [7,16]. Further studies using IFNAR1-deficient cells may help elucidate additional mechanisms by which the LOS core contributes to increased pathogenicity.

Studies have explored the potential development of novel drugs and vaccines targeting the LPS-TLR4 signaling pathway. TLR4 activators are potential therapeutic immunomodulators and adjuvants [37]. LPS neutralization and TLR4 blockade are also promising therapeutic strategies [38]. We showed, in this study, that using the LOS core region as a vaccine candidate or targeting the LOS core, its synthetic enzymes, and the interface between the LOS core and CD14 to develop drugs or antibodies may potentially aid in combating *A. baumannii*.

This study has some limitations. While enhanced CD14-associated TLR4 endocytosis increases IFN-β production [39,40], the mechanism by which LOS enhances CD14 endocytosis remains unclear. Other receptors, such as lectins, may recognize the saccharide components of LPS and contribute to CD14-associated TLR4 endocytosis. Moreover, the molecular structural differences between LOS and Re-LOS and their interactions with CD14 were lacking in this study. Further structural biology techniques, such as X-ray crystallography, molecular docking, nuclear magnetic resonance spectroscopy, and mass spectrometry, should be considered to provide a more detailed structural characterization of the interactions between LOS and CD14.

## Conclusion

Our study highlights the pivotal role of the core region of *A. baumannii* LOS in enhancing TLR4 endocytosis, leading to increased IFN-β production and virulence. These findings could influence the development of novel therapeutic strategies targeting the LOS core to effectively combat *A. baumannii* infections.

## Materials and methods

### Ethics statement

The protocols of the study were approved by the Laboratory Animal Center of the National Defense Medical University (IACUC-21–33 and IACUC-22–236).

### Bacterial strains

Bacterial strains used in this study are listed in S3 Table. The multi-locus sequence typing scheme involving polymerase chain reaction (PCR) amplification and sequencing of seven housekeeping genes (*gltA, gyrB, gdhB, recA, cpn60, gpi, and rpoD*) was performed on the selected isolates as previously described [41]. Strains with Re-LOS were constructed as previously described [42,43]. The *lpsB* gene in *A. baumannii* genomic DNA was disrupted by inserting an apramycin antibiotic resistance gene (*Apr*^R^) to generate knockout mutants. Briefly, the *Apr*^R^ integration cassette was obtained with PCR amplification from the pGEM-T-Easy Vector DNA template using P1 and P2 primers. Approximately 500 bp regions in R1 and R2, corresponding to the 5′-proximal and 3′-proximal regions of the *lpsB* coding sequence, were amplified using P3/P4 (R1 region) and P5/P6 (R2 region) primers. The three PCR products were subsequently combined (S1a Fig) and reamplified to generate a linear fragment containing an apramycin resistance cassette flanked by two specific regions. The linear product was cloned into a pGEM-T Easy Vector. The plasmid was taken up into *A. baumannii* competent cells by electroporation and subsequently selected on Luria-Bertani (LB) agar plates supplemented with 50 μg/mL apramycin. The successful generation of these *ΔlpsB* mutants carrying Re-LOS was verified using PCR (S1c Fig). The primer sequences are listed in S4 Table. To complement the mutant phenotype, the wild-type *lpsB* gene was cloned into an *A. baumannii*-*E. coli* shuttle vector pAbYm2 [44] to generate pAbYm2*lpsB*. The shuttle vector pYMAb2 was previously generated in our lab and derived from *E. coli* plasmid pET-28a, which has a kanamycin resistant determinant and an SphI/XbaI fragment containing replicons from *A. baumannii* ATCC19606 plasmid pMAC [44]. The vector pAbYm2*lpsB* was electroporated into the *ΔlpsB* mutant to construct the complement strain, *ΔlpsB*::*lpsB*. The successful generation of these mutants was verified using PCR.

To better display the presence and absence of the LOS in the bacterial strains. Two *E. coli* keio knockout strains were purchased from Horizon discovery (Cambridge, UK) and used for the silver staining of LOS. *E. coli* BW25113 lacking *waaL* gene (Ec*ΔrfaL*) produces lipopolysaccharides deficient in the O-antigen was used as a reference of *Ab908*. In addition, *E. coli* BW25113 lacking *waaC* gene (Ec*ΔrfaC*) produces truncated lipopolysaccharides, specifically losing the outer core and parts of the inner core was used as a reference of *Ab908*Δ*lpsB* [45].

### Silver staining of LOS

LOS from *A. baumannii* and *E. coli* were extracted using an LPS extraction kit (Boca Scientific Inc., Waltham, MA, USA). The extracted samples were treated with RQ1 RNase-Free DNase (Promega, Madison, WI, USA) and RNase A (25 μg/mL; QIAGEN, Hilden, Germany) at 37 °C for 2 h to remove contaminating nucleic acids, followed by incubation with proteinase K (100 μg/mL; Sigma**–**Aldrich, St. Louis, MO, USA) at 37 °C overnight to eliminate residual proteins. The samples were then mixed with 4 × Laemmli sample buffer (Bio-Rad, Hercules, CA, USA) and heated at 95 °C for 5 min prior to electrophoresis. Purified LOS were separated on 20% sodium dodecyl sulfate–polyacrylamide gels (SDS-PAGE) and visualized using a Pierce Silver Stain kit (Thermo Fisher Scientific, Waltham, MA, USA) according to the manufacturer’s instructions.

Briefly, gels were washed twice with ultrapure water (5 min each), fixed twice in 30% ethanol and 10% acetic acid (15 min each), and then oxidized in a solution containing 1% periodic acid in 30% ethanol and 10% acetic acid for 20 min. The gels were subsequently washed twice in 10% ethanol (5 min each), followed by three washes in ultrapure water (5 min each). For sensitization, gels were treated with sensitizer solution for 1 min and washed twice. Gels were then stained with a mixture of enhancer and stain solution for 30 min and developed using enhancer and developer solution for 5 min. The reaction was terminated by incubation in 5% acetic acid for 10 min.

### Growth of the isolates

Briefly, 10 mL of LB broth was inoculated with a 100 μL overnight culture and incubated at 37 °C with shaking at 200 revolutions per min (rpm) for 6 h. Aliquots were obtained at 0, 1, 2, 4, 6, and 24 h post-inoculation, serially diluted, and plated on trypticase soy agar (TSA) plates to determine the viable cell counts.

### Cell lines and culture conditions

Human THP1-Dual, THP1-Dual KO‑TRIF, and THP1-Dual KO-MyD88 were purchased from InvivoGen (San Diego, CA, USA). J774a.1 murine macrophage cells were purchased from Sigma**–**Aldrich (St. Louis, MO, USA). The human THP1-Dual, THP1-Dual KO‑TRIF, and THP1-Dual KO-MyD88 cells were derived from the Human THP1 monocyte cell line by stable integration of an IRF-luciferase reporter and NF-κB alkaline phosphatase (secreted embryonic alkaline phosphatase, SEAP) reporter. The cells were used for simultaneous study of the NF-κB pathway, by monitoring the activity of SEAP, and the IRF pathway, through assessment of the activity of Lucia luciferase.

All the cells were maintained in Roswell Park Memorial Institute (RPMI) 1640 medium supplemented with 10% heat-inactivated fetal bovine serum, 25 mM HEPES, 2 mM L-glutamine, 100 μg/mL Normocin, and Penicillin-Streptomycin solution (100 U/mL-100 μg/mL). Cultures were incubated in a humidified incubator at 37 °C using 5% carbon dioxide.

To differentiate the macrophage phenotype, THP1-Dual, THP1-Dual KO-TRIF, and THP1-Dual KO-MyD88 cells were treated with phorbol 12-myristate 13-acetate (PMA) at a concentration of 50 ng/mL in cell culture plates (approximately 5 × 10^6^ cells/mL) for 24 h [46]. Following incubation, PMA was removed.

### Chemical sources

Triton X-100, polymyxin B, L48H37 (MD-2 inhibitor), and U-73122 (PLCγ2 inhibitor) were purchased from Sigma**–**Aldrich (St. Louis, MO, USA). DNase I and RNase A were purchased from QIAGEN (Hilden, Germany). TAK242 (TLR4 inhibitor) was purchased from Merck (Darmstadt, Germany). Dynasore (endocytosis inhibitor) was purchased from Abcam (Cambridge, UK). Piceatannol (a Syk inhibitor) was purchased from Bio-Techne (Minneapolis, MN, USA). MRT67307 (IKKε/TBK1 inhibitor), hIgG (anti-human IgG), and hCD14 (purified anti-human CD14 antibody) were purchased from InvivoGen (San Diego, CA, USA). The anti-IFN-β antibody and mouse IgG2a (C1.18.4) isotype control antibody for animal experiments were purchased from Ichorbio (Wantage, UK). The Cell Counting Kit (CCK kit) was purchased from DOJINDO Laboratories (Kumamoto, Japan).

### Animal experiments

Wild-type C57BL/6 mice were purchased from the National Laboratory Animal Center (NLAC, Taipei, Taiwan). The IFNAR1-deficient mice (Ifnar1-/-) were kindly provided by Dr. Guann-Yi Yu (National Health Research Institutes, Taiwan). All the mouse experiments were conducted following the recommendations of the Laboratory Animal Center of the National Defense Medical University. The ARRIVE guidelines were adhered to for all mouse experiments, and the protocols were approved by the Laboratory Animal Center of the National Defense Medical University (IACUC-21–33 and IACUC-22–236). The experiments were carefully designed to minimize the number of animals used while ensuring statistical significance. Appropriate anesthesia and analgesia were administered to alleviate potential discomfort during the procedure.

### Isolation and purification of MVs

MVs were isolated from late log phase cultures (16 h) of *A. baumannii*. Briefly, 400 mL of LB broth was inoculated with 4 mL of an overnight culture and incubated at 37 °C with shaking at 200 rpm for 6 h. The cells were pelleted by centrifuging at 12,250 × g for 20 min, and the supernatant was filtered through a 0.22 μm membrane filter (JET BIOFIL, China) to remove cells and cellular debris. The filtrate was subjected to ultracentrifugation at 142,000 × g for 2 h at 4 °C using a Type 45 Ti rotor (Beckman, USA). To wash the MVs, the pellet was resuspended in Phosphate-buffered saline (PBS) and ultracentrifuged again at 142,000 × g for 2 h at 4 °C using the same rotor. The final pellet was resuspended in 800 μL of PBS and kept on ice. The resulting MV suspension was cultured on Mueller-Hinton agar and confirmed to be free of bacteria.

### Characterization of MVs using a nanoparticle tracking analyzer

The nanoparticle tracking analyzer (NTA) device, ZetaView PMX120 (Particle Metrix GmbH, Meerbusch, Germany), was used to measure the size distribution of MVs at 25 °C. Polystyrene standard beads (Thermo Fisher Scientific, Waltham, MA, USA) were used to align the focus and camera/laser positions. Samples were diluted in PBS to 1 mL and measured at 25 °C. Each sample was scanned at 11 cell positions, and particle sizes were calculated based on their Brownian motion. Data were processed using ZetaView software v8.05.10. Measurements were performed in scatter mode using a 488 nm laser, while fluorescence was detected in fluorescence mode using the same laser with a 500 nm long-pass filter. All experiments were performed in triplicate.

### Mice pneumonia model

Seven-week-old female C57BL/6 mice were injected intratracheally with strains of *A. baumannii*. The bacterial inoculum (colony-forming units, CFU) was determined from aliquots of suspended bacteria that were serially diluted and plated onto TSA for enumeration. In the first experiment, mice were monitored every 12 h for 72 h after bacterial exposure for signs of morbidity. The mice were sacrificed, and the lungs were harvested and weighed. In the second experiment, mice were euthanized 4 h after bacterial exposure. The lungs were harvested, homogenized, and plated on TSA to enumerate bacterial counts. Blood samples were collected and subjected to differential white blood cell counts. The serum and BALF of mice were obtained 4 h after bacterial exposure and sent for cytokine and chemokine determination in the third experiment.

### Mice intraperitoneal sepsis model

Three experiments were performed using a mouse model of intraperitoneal sepsis. In the first experiment, seven-week-old female C57BL/6 mice were instilled intraperitoneally with MVs (~8.6 × 10^11^ particles in 100 μL) from different *A. baumannii* strains. Mice were monitored for signs of morbidity every 12 h for 96 h after bacterial exposure. In the second experiment, 7-week-old female C57BL/6 and IFNAR1-deficient mice (Ifnar1-/-) were instilled intraperitoneally with MVs (~9.7 × 10^11^ particles in 100 μL) from Ab908 and Ab908*ΔlpsB*. The mice were monitored for signs of morbidity every 12 h for 96 h after MVs instillation. In the third experiment, 7-week-old female C57BL/6 mice were instilled intraperitoneally with MVs (~9 × 10^11^ particles in 100 μL) from Ab908. The anti-IFN-β antibody (300 μg or 600 μg) or IgG2a (C1.18.4) isotype control antibody (600 μg) was instilled intraperitoneally 30 min later. Mice were observed for signs of morbidity every 12 h for 96 h.

### Enzyme-linked immunosorbent assay for cytokines

THP1-Dual or J774a.1 cells were seeded in a 24-well plate at a density of 5 × 10^5^ cells/well and cultured overnight with 50 ng/mL PMA. The following day, cells were washed with PBS and cultured in fresh RPMI 1640 medium overnight. For inhibition experiments, cells were pre-treated with the following inhibitors at the specified concentrations and durations: DNase I (1 U/μL), RNase A (200 μg/mL), polymyxin B (3 μM), K (100 μg/mL), L48H37 (10 μM), piceatannol (75 μM), and U73122 (5 μM) for 0.5 h; TAK242 (1 μM), hCD14 (10 μg/mL), hIgG (10 μg/mL), dynasore (80 μM), and MRT67307 (10 μM) for 1 h. After pre-treatment, the cells were stimulated with MVs. Subsequently, the supernatants were collected 4 h after stimulation, and cytokine levels were measured using enzyme-linked immunosorbent assay (ELISA) kits (R&D Diagnostics, Minneapolis, MN, USA).

### RNA extraction and quantitative real-time polymerase chain reaction

Quantitative Real-time polymerase chain reaction was used to determine cytokine gene expression levels. Briefly, macrophages were stimulated with MVs for 4 h, and total RNA was extracted using the Qiagen RNeasy Mini Kit (Qiagen, Netherlands) following the manufacturer’s instructions. cDNA was generated by the reverse transcription of 1–2 μg of total RNA using oligo dT primers and M-MuLV reverse transcriptase in a total reaction volume of 20 μL (Thermo Fisher Scientific, Waltham, MA, USA). Gene transcripts were quantified using the Power SYBR Green PCR Master Mix (Applied Biosystems, Waltham, MA, USA) on a QuantStudio 3 Real-Time PCR System (Thermo Fisher Scientific, Waltham, MA, USA). Amplification specificity was evaluated using melting curve analysis. The primer sequences are listed in S4 Table.

### RNA sequencing and data analysis

J774a.1 cells were seeded in plates and subsequently stimulated with MVs of Ab908 and Ab908Δ*lpsB* (MOI = 500) for 2 h. The cells were harvested at the end of stimulation, which was followed by RNA extraction. The purity and quantity of RNA were checked using a SimpliNanoBiochrom spectrophotometer (Biochrom, MA, USA). RNA degradation and integrity were monitored using a Qsep 100 DNA/RNA Analyzer (BiOptic Inc., Taiwan). One microgram of total RNA per sample was used as input material for the RNA sequencing sample preparations. Sequencing libraries were generated using the KAPA mRNA HyperPrep Kit (KAPA Biosystems, Roche, Basel, Switzerland), following the manufacturer’s recommendations. The index codes were added for the unique identification of each sample. The sequences were determined using the Illumina NovaSeq 6000 platform. Sequencing data (FASTQ reads) were generated using bcl2fastq v2.20, followed by adaptor clipping and sequence quality trimming using Trimmomatic (v0.36) [47]. Filtered reads were mapped to mm10 using HISAT2 software [48]. Genes in the mapped reads were identified using featureCounts [49]. DEGs, GSEA biological processes, and TF-binding motifs were analyzed using integrated Differential Expression and Pathway analysis [50].

### Biological activity

QUANTI-Luc (rep-qlc1) was used to detect the level of luciferase by adding it to the culture supernatant and reading immediately using a plate reader (SpectraMax iD5; Molecular Devices, California, CA, USA) at a 0.15-s reading time. QUANTI-Blue (rep-qb1) was used to detect the level of SEAP by adding it to the culture supernatant, incubating for 30 min, and measuring the absorbance with a plate reader (SpectraMax iD5) at 620 nm. Quanti Luc and Quanti Blue were purchased from InvivoGen (San Diego, CA, USA)

### Cytotoxicity of the inhibitors

The CCK kit was used to evaluate the cytotoxicity of the inhibitors following the manufacturer’s recommendations. Briefly, J774a.1 cells were grown in 96-well plates at 12,500 cells/well in Dulbecco’s Modified Eagle Medium (DMEM). Overnight cell cultures were washed and replaced with fresh DMEM. The cells were pretreated with the indicated concentrations of the inhibitors for 1 h. Wells containing DMEM alone were used as controls. MVs of the three isolates were added to 96-well plates. Following a 6-h exposure to the MVs, CCK solution was added to each well, and the plates were re-incubated for 1 h. Finally, 10 μL 0.1 M HCl was added to each well to stop the reaction. The number of viable cells was assessed by measuring absorbance at 450 nm using a microplate spectrophotometer.

### Western blot analysis

J774a.1 cells were seeded in 6-well (1 × 10^7^ cells/mL) plates, followed by adding 1 µg/mL LPS and MVs. After incubation for 1 h or 6h, the total proteins were extracted using a protein extraction kit [51]. Protein concentrations were measured using a Bradford Protein Assay kit. Following this, equal amounts of protein (50 µg) from each sample were heated to 95˚C for 5 min with 4 × Laemmli sample buffer and subsequently separated using SDS-PAGE (10% gel). Proteins were transferred onto polyvinylidene fluoride membranes. Following blocking for 1 h with protein-free blocking buffer at room temperature, the membranes were incubated with primary antibodies against GAPDH, IRF3, and phosphorylated IRF3 overnight at 4˚C. Following washing three times with Tris-Buffered Saline with Tween-20 buffer, the membranes were incubated with the aforementioned secondary antibodies for 1 h at room temperature. The electrochemiluminescence kit and GAPDH antibody were purchased from GeneTex International Corporation (Hsinchu, Taiwan). The phospho-IRF-3 antibody was purchased from Cell Signaling Technology (Danvers, MA, USA). The IRF3 antibody was purchased from Abcam (Cambridge, UK).

### Flow cytometry for TLR4 endocytosis and TLR4/MD-2 dimerization

TLR4 endocytosis and TLR4/MD-2 dimerization were measured using flow cytometry [24]. Briefly, J774a.1 cells were seeded in well plates and stimulated as previously indicated at 37 °C. The cells were subsequently washed and stained with the appropriate antibodies, and bovine serum albumin was used to reduce nonspecific binding of the antibodies. The surface receptors were stained using a BD FACSCanto II. The mean fluorescence intensities (MFIs) of CD14 and TLR4 in unstimulated and stimulated cells were recorded. The percentage of TLR4/MD-2 dimer formation was calculated as follows: 100%－the percentage of TLR4/MD-2 monomer formation; this percentage was determined as the ratio of the MFI values of stimulated cells to those of unstimulated cells.

### Statistical analysis

For continuous variables, the Student’s t-test was used to assess differences. Time to mortality was analyzed using the Kaplan–Meier survival analysis and the log–rank test. All analyses were processed using SPSS version 26.0.

## Supporting information

S1 Table 1. The size of membrane vesicles from different strains used in this study. (DOCX)

S2 Table 2. Enriched transcription factor (TF) binding motifs in promoters of differentially expressed genes (DEGs). (DOCX)

S3 Table Bacterial isolates used in this study. (DOCX)

S4 Table The primers used in this study. (DOCX)

S1 Fig The structure of the plasmid, the PCR mapping for the confirmation of successful deletion of *lpsB gene,* the lipooliosaccharide (LOS) structure, and the growth of the bacterial strains used in this study. Schematic presentation of the plasmid used for the generating the *lpsB* gene knockout *Acinetobacter baumannii* mutant **(a)**. Schematic presentation of the *A. baumannii* strains harboring different length of LOS **(b)**. PCR mapping for the confirmation of successful deletion of *lpsB* gene **(c)**. Silver stain of the purified LOS shows that *A. baumannii* 908 (Ab908), the complementary strain (Ab908Δ*lpsB*::*lpsB*), and *Escherichia coli* BW25113 lacking *waaL* gene (*EcΔrfaL*) produce LOS with core, but not the *lps*B deletion mutant strains (Ab908Δ*lpsB*) and *EcΔrfaC* **(d)**. Growth curve of the three *A. baumannii* strains **(e)**.
(TIF)

S2 Fig RNA sequencing analysis of the gene expression. RNA sequencing analysis showed differential gene expression after stimulation with membrane vesicles (MVs) from the wild-type *A. baumannii* strain Ab908 (harboring intact LOS) and Ab908*ΔlpsB* (harboring Re-LOS) **(a)**. Volcano plots depicting gene expression changes in J774a.1 cells, showing fold change and *P* value comparisons after stimulation with MVs from Ab908 and Ab908*ΔlpsB* **(b)**.
(TIF)

S3 Fig Cytotoxicity of the inhibitors used in this study. The J774a.1 cells were grown in 96-well plates at a density of 12,500 cells per well and treated with inhibitors with indicated concentrations for 1 h. The membrane vesicles (MVs) of three different isolates were then added to the 96-well plates. After 6 h exposure to the MVs, the number of viable cells was assessed by measurement of the absorbance at 450 nm using a microplate spectrophotometer. The concentration of the inhibitors used were as following: DNase I, 1 U/μL; RNAse A, 200 μg/mL; polymyxin B, 3 μM; TAK242, (TLR4 inhibitor, 1 μM); L48H37, (MD-2 inhibitor, 10 μM); MRT67307 (IKKε/TBK1 inhibitor, 10 μM), dynasore (endocytosis inhibitor, 80 μM), piceatannol (Syk inhibitor, 75 μM), U-73122 (PLCγ2 inhibitor, 5 μM); PBS, phosphate-buffered saline.
(TIF)
