# Supplementary material for: Acinetobacter baumannii lipooligosaccharide core region promotes CD14-dependent TLR4 endocytosis and enhances pathogenicity through interferon-β production
Source: PLoS Pathog. 2026 Jul 14;22(7):e1014364. doi: 10.1371/journal.ppat.1014364 (PMC13367702; doi:10.1371/journal.ppat.1014364)
Supplement: S1 Table — (DOCX) [file ppat.1014364.s001.docx]

**S1 Table. The size of membrane vesicles from different strains used in this study.**

| Strains | Size (nm) |
| --- | --- |
| Ab908 | 159.4 ± 4.5 |
| Ab908*ΔlpsB* | 189.9 ± 11.4 |
| Ab908*ΔlpsB*::*lpsB* | 162.8 ± 4.4 |
