# Supplementary material for: Acinetobacter baumannii lipooligosaccharide core region promotes CD14-dependent TLR4 endocytosis and enhances pathogenicity through interferon-β production
Source: PLoS Pathog. 2026 Jul 14;22(7):e1014364. doi: 10.1371/journal.ppat.1014364 (PMC13367702; doi:10.1371/journal.ppat.1014364)
Supplement: S2 Table — (DOCX) [file ppat.1014364.s002.docx]

**S2 Table. Enriched transcription factor (TF) binding motifs in promoters of differentially expressed genes (DEGs).**

| List | Motif | TF | TF family | FDR |
| --- | --- | --- | --- | --- |
| Down regulated | TAA | Isl2 | Homeodomain | 0.0E+00 |
|  | TTCATTGA | Bbx | Sox | 0.0E+00 |
|  | CCACGTG | Mycl1 | bHLH | 0.0E+00 |
|  | CCATATG | Neurod2 | bHLH | 0.0E+00 |
|  | TTTGTCATGTAG | Irx3 | Homeodomain | 0.0E+00 |
|  | TCACGTG | Usf2 | bHLH | 2.4E-03 |
|  | AGGATGATGCAATC | Atf4 | bZIP | 6.5E-03 |
|  | CCATATGT | Twist2 | bHLH | 8.8E-03 |
|  | AGGTGTGA | Tbx4 | T-box | 8.1E-02 |
|  | TCGCGTGA | Srebf1 | bHLH | 2.0E-01 |
| Up regulated | GAAAATGAAACTGA | Stat2 | STAT | 3.7E-13 |
|  | AACGAAA | Irf2 | IRF | 6.1E-12 |
|  | AAAGTGAAAGTGAAAGT | Irf1 | IRF | 1.5E-11 |
|  | GAAAGTGAAAGT | Prdm1 | C2H2 ZF | 5.4E-11 |
|  | GAAAC | Irf3 | IRF | 9.4E-11 |
|  | CGAAAC | Irf5 | IRF | 2.2E-10 |
|  | GAAAC | Irf4 | IRF | 4.1E-10 |
|  | GAAA | Irf9 | IRF | 9.1E-10 |
|  | CGAAAC | Irf6 | IRF | 6.8E-09 |
|  | AAACGAAA | Irf7 | IRF | 3.0E-08 |
|  | GGAA | Elf3 | Ets | 5.8E-05 |
|  | TAAACA | Foxo4 | Forkhead | 6.4E-05 |
|  | TAAACAAA | Foxj1 | Forkhead | 1.5E-04 |
|  | GGAA | Elf3 | Ets | 1.9E-04 |
|  | TGACTCAGCA | Nfe2 | bZIP | 2.4E-04 |
|  | AACA | Foxo6 | Forkhead | 2.4E-04 |
|  | TTTCCA | Nfatc3 | Rel | 3.1E-04 |
|  | AAACA | Foxg1 | Forkhead | 5.2E-04 |
|  | TTTCCA | Nfatc2 | Rel | 6.8E-04 |
|  | AACCGGAAAT | Etv2 | Ets | 8.1E-04 |

FDR, False discovery rate.
