# Supplementary material for: Acinetobacter baumannii lipooligosaccharide core region promotes CD14-dependent TLR4 endocytosis and enhances pathogenicity through interferon-β production
Source: PLoS Pathog. 2026 Jul 14;22(7):e1014364. doi: 10.1371/journal.ppat.1014364 (PMC13367702; doi:10.1371/journal.ppat.1014364)
Supplement: S3 Table — (DOCX) [file ppat.1014364.s003.docx]

**S3. Table. Bacterial isolates used in this study.**

| Strain no | MLST type | Characteristics | Descriptions | |
| --- | --- | --- | --- | --- |
| Ab908 | ST208 | A clinical carbapenem-resistant *A. baumannii* isolate recovered from a patient with bloodstream infection in 2004. | Resistant to amikacin, ceftazidime, cefepime, ciprofloxacin, piperacillin-tazobactam, sulbactam, and imipenem. |  |
| Ab908Δ*lpsB* | ST208 | *A. baumannii* 908 lacking *lpsB* gene. *lpsB* gene was deleted by double crossover method. The success deletion was confirmed by PCR. | Resistant to amikacin, ceftazidime, cefepime, ciprofloxacin, piperacillin-tazobactam, sulbactam, and imipenem. |  |
| Ab908Δ*lpsB*::*lpsB* | ST208 | *A. baumannii* 908Δ*lpsB* containing complemented *lpsB* gene. | Resistant to amikacin, ceftazidime, cefepime, ciprofloxacin, piperacillin-tazobactam, sulbactam, and imipenem. |  |
| Ab1033 | ST218 | A clinical carbapenem-resistant *Acinetobacter baumannii* isolate recovered from a patient with bloodstream infection in 2004. | Resistant to amikacin, ceftazidime, cefepime, ciprofloxacin, piperacillin-tazobactam, sulbactam, and imipenem. |  |
| Ab1033Δ*lpsB* | ST218 | *A. baumannii* 1033 lacking *lpsB* gene. | Resistant to amikacin, ceftazidime, cefepime, ciprofloxacin, piperacillin-tazobactam, sulbactam, and imipenem. |  |
| Ab2804 | ST473 | A clinical carbapenem-resistant *Acinetobacter baumannii* isolate recovered from a patient with bloodstream infection in 2008. | Resistant to amikacin, ceftazidime, cefepime, ciprofloxacin, piperacillin-tazobactam, sulbactam, and imipenem. |  |
| Ab2804Δ*lpsB* | ST473 | *A. baumannii* 2804 lacking *lpsB* gene. | Resistant to amikacin, ceftazidime, cefepime, ciprofloxacin, piperacillin-tazobactam, sulbactam, and imipenem. |  |
| Ec*ΔrfaL* | -- | *E. coli* BW25113 lacking *waaL* gene produces lipopolysaccharides deficient in the O-antigen. | An *E. coli* K-12 *waaL* gene knockout mutant strain used as a reference of Ab908 for SDS-PAGE in this study. |  |
| Ec*ΔrfaC* | -- | *E. coli* BW25113 lacking *waaC* gene produces truncated lipopolysaccharides, specifically losing the outer core and parts of the inner core. | An *E. coli* K-12 *waaC* gene knockout mutant strain used as a reference of Ab908Δ*lpsB* for SDS-PAGE in this study. |  |
