# Supplementary material for: Acinetobacter baumannii lipooligosaccharide core region promotes CD14-dependent TLR4 endocytosis and enhances pathogenicity through interferon-β production
Source: PLoS Pathog. 2026 Jul 14;22(7):e1014364. doi: 10.1371/journal.ppat.1014364 (PMC13367702; doi:10.1371/journal.ppat.1014364)
Supplement: S4 Table — (DOCX) [file ppat.1014364.s004.docx]

**S4 Table. The primers used in this study.**

| **Primers** | **Sequences** | | **Location or Target gene** | |
| --- | --- | --- | --- | --- |
| **Primers used for knockout and confirmation of the *lpsB* gene** | | | | |
| P1 | | 5’-GTCTACACGAACCCTTTGGCAA-3’ | | *Apra^R^* |
| P2 | | 5’-TGGAACTTATGAGCTCAGCCAA-3’ | | *Apra^R^* |
| P3 | | 5’-ATGAAAGTGATGCAACTTCT-3’ | | Upstream of *lpsB* |
| P4 | | 5’-GGTTCGTGTAGACGACTTGGTTAAACCACTGT-3’ | | Upstream of *lpsB* |
| P5 | | 5’-GCTCATAAGTTCCAACTTTGCCTACCT-3’ | | Downstream of *lpsB* |
| P6 | | 5’-TCAATTCAATACACTTTGAT-3’ | | Downstream of *lpsB* |
| P7 | | 5’-AACGCAACAGCGACATCCATGT-3’ | | Flanking of *lpsB* |
| P8 | | 5’-GTAGGTAACGCGCTTGCTGTACTT-3’ | | Flanking of *lpsB* |
| P9 | | 5’-TGTAAAACGACGGCCAGT-3’ | | pGEM T vector M13 forward sequencing primer binding site |
| P10 | | 5’-CGTATATCTGAGCGATGTC-3’ | | Downstream of *lpsB* |
| **Primers used for qRT-PCR** | | | |  |
| RT-GAPDH-F(169) | 5’-TCACCACCATGGAGAAGGC-3’ | | | *GAPDH* |
| RT-GAPDH-R(169) | 5’-GCTAAGCAGTTGGTGGTGCA-3’ | | | *GAPDH* |
| RT-IFN-β-F(314) | 5’-TCCAAGAAAGGACGAACATTCG-3’ | | | *IFN-β* |
| RT-IFN-β-R(314) | 5’-TGAGGACATCTCCCACGTCAA-3’ | | | *IFN-β* |
| RT-CXCL9-F(188) | 5’-CTTGAGCCTAGTCGTGATAAC-3’ | | | *CXCL9* |
| RT-CXCL9-R(188) | 5’-CCAGCTTGGTGAGGTCTATC-3’ | | | *CXCL9* |
| RT-CXCL10-F(130) | 5’-TGAGATCATTGCCACGAT-3’ | | | *CXCL10* |
| RT-CXCL10-R(130) | 5’-GCTTCACTCCAGTTAAGG-3’ | | | *CXCL10* |
| RT-CXCL11-F(151) | 5’-AGATCCAAGCAAGCTCGCCTCATA-3’ | | | *CXCL11* |
| RT-CXCL1-R(151) | 5’-ATGTTCCAAGACAGCAGAGGGTCA-3’ | | | *CXCL11* |
| RT-Rsad2-F(194) | 5’-AACAGGCTGGTTTGGAGAAGA-3’ | | | *Rsad2* |
| RT-Rsad2-R(194) | 5’-AGCAAGAATGTCCAAATACTCCC-3’ | | | *Rsad2* |

^#^ The sequences shown in bold represent the sequence of *lpsB* gene.
