# Supplementary material for: Acinetobacter baumannii lipooligosaccharide core region promotes CD14-dependent TLR4 endocytosis and enhances pathogenicity through interferon-β production
Source: PLoS Pathog. 2026 Jul 14;22(7):e1014364. doi: 10.1371/journal.ppat.1014364 (PMC13367702; doi:10.1371/journal.ppat.1014364)
Supplement: Attachment — Submitted filename: Response to Reviewers 20250919.docx [file ppat.1014364.s009.docx]

**Response letter**

**Manuscript ID: PPATHOGENS-D-25-00850
*Acinetobacter baumannii* lipooligosaccharide core region enhances CD14-dependent TLR4 endocytosis and boosts virulence through interferon-β production**

Dear Editor and Reviewers,

I hope this message finds you well. Thank you for the opportunity to revise and resubmit our manuscript entitled “***Acinetobacter baumannii* lipooligosaccharide core region enhances CD14-dependent TLR4 endocytosis and boosts virulence through interferon-β production**”. We are grateful for the insightful comments provided by the reviewers, which have helped us improve the quality and clarity of our work.

We have carefully addressed each of the reviewers’ comments in the revised manuscript and have provided a detailed point-by-point response. Specifically:

- We modified the title of the manuscript to “*Acinetobacter baumannii* lipooligosaccharide core region promotes CD14-4 dependent TLR4 endocytosis and enhances pathogenicity through interferon-β production*”* to make it more suitable for the findings of the research.
- Experiments involving membrane vesicles (MVs) have been normalized based on particle number, as assessed by nanoparticle tracking analysis.
- Given that further work is needed to conclusively determine the affinity of CD14 for different LOS variants, we have softened the wording related to this aspect accordingly.
- Additional experiments, including in animal studies in Fig. 6, have been performed to strengthen and support our conclusions.
- GAPDH loading controls for the 1 h and 6 h samples have been added.
- Cell viability after treatment of the inhibitors had been tested and confirmed.
- The Material and Methods section has been expanded to include all missing details, including bacterial inoculum (CFU), reagent and inhibitor concentrations, and complete experimental procedures.
- We have added Fig 7 as the schematic figure to help clarify the major finding of the study.

We believe that these revisions significantly improve the manuscript and adequately address the concerns raised by the reviewers. We have submitted both the revised manuscript and a point-by-point response for your consideration. Please do not hesitate to contact us should any further clarification be required.

Thank you once again for your time and consideration.

Warm regards,

Yung-Chih Wang

Division of Infectious Disease and Tropical Medicine, Department of Internal Medicine, Tri-Service General Hospital, National Defense Medical Center

7F, No. 325, Section 2, Cheng-Kung Road, Neihu 114, Taipei, Taiwan

Tel: +886-2-87927257

Fax: +886-2-87927258

Email: [wystwyst@gmail.com](mailto:wystwyst@gmail.com)

**Additional Editor Comments:**
Three experts in the topic have revised this manuscript. The three of them found the work interesting. However, they have raised concerns that need to be addressed in order to fully support the authors' conclusions. In particular, the work with OMV needs more quantifications/controls. Visualization by microscopy and proper LOS/protein quantifications are needed. The affinity of CD14 to the different LOS needs to be investigated by other methods. In Figure 4e, the GAPDH loading controls must be shown for the 1 h samples and the 6 h samples. For all experiments using inhibitors, controls showing maintenance of viability or secretion of other cytokines/chemokines are needed. E.g. Fig 3b, Fig 3c, Fig 4f, Fig 4i, Fig. 5a. Finally, there are concerns about the results in Figure 6 that require more work to be conclusive. Details pm experimental procedures including bacterial inoculum (CFU), reagents and inhibitors concentration, as well as complete procedures are missing from Material and Methods.

**A: We are grateful for the insightful comments provided by the editor and reviewers. Below are our responses to the editor’s comments.**

- Experiments involving membrane vesicles (MVs) have been normalized based on particle number, as assessed by nanoparticle tracking analysis.
- Given that further work is needed to conclusively determine the affinity of CD14 for different LOS variants, we have softened the wording related to this aspect accordingly.
- Additional experiments, including in animal studies in Fig. 6, have been performed to strengthen and support our conclusions.
- GAPDH loading controls for the 1 h and 6 h samples have been added.
- Cell viability after treatment of the inhibitors had been tested and confirmed.
- The Material and Methods section has been expanded to include all missing details, including bacterial inoculum (CFU), reagent and inhibitor concentrations, and complete experimental procedures.
- We have added Fig 7 as the schematic figure to help clarify the major finding of the study.

**Journal Requirements:**

1) Please ensure that the CRediT author contributions listed for every co-author are completed accurately and in full.
At this stage, the following Authors/Authors require contributions: Yi-Tzu Lee, Te-Li Chen, Shu-Chen Kuo, Yu-Han Liu, Meng-Chang Lee, Ming-Fen Chuang, Mei-Chei Tan, I-Ming Lee, Ya-Sung Yang, and Yung-Chih Wang. Please ensure that the full contributions of each author are acknowledged in the "Add/Edit/Remove Authors" section of our submission form.
The list of CRediT author contributions may be found here: https://journals.plos.org/plospathogens/s/authorship#loc-author-contributions

A: The CRediT author contributions listed for every co-author are completed accurately and in full.

2) We do not publish any copyright or trademark symbols that usually accompany proprietary names, eg ©,  ®, or TM  (e.g. next to drug or reagent names). Therefore please remove all instances of trademark/copyright symbols throughout the text, including:
- TM on pages: 32, 33, 34, and 35.

A: The copyright or trademark symbols were removed.

3) We have noticed that you have uploaded Supporting Information files, but you have not included a list of legends. Please add a full list of legends for your Supporting Information files after the references list.

A: The full lists of legends for supporting information files were provided.

4) Some material included in your submission may be copyrighted. According to PLOSu2019s copyright policy, authors who use figures or other material (e.g., graphics, clipart, maps) from another author or copyright holder must demonstrate or obtain permission to publish this material under the Creative Commons Attribution 4.0 International (CC BY 4.0) License used by PLOS journals. Please closely review the details of PLOSu2019s copyright requirements here: PLOS Licenses and Copyright. If you need to request permissions from a copyright holder, you may use PLOS's Copyright Content Permission form.
Please respond directly to this email and provide any known details concerning your material's license terms and permissions required for reuse, even if you have not yet obtained copyright permissions or are unsure of your material's copyright compatibility. Once you have responded and addressed all other outstanding technical requirements, you may resubmit your manuscript within Editorial Manager. 
Potential Copyright Issues:
i) Figures 1A, 1I, 5E, and S1B. Please confirm whether you drew the images / clip-art within the figure panels by hand. If you did not draw the images, please provide (a) a link to the source of the images or icons and their license / terms of use; or (b) written permission from the copyright holder to publish the images or icons under our CC BY 4.0 license. Alternatively, you may replace the images with open source alternatives. See these open source resources you may use to replace images / clip-art:
- https://commons.wikimedia.org
- https://openclipart.org/.

A: There are no copyright issues. All the images were drawn by hand.

5) Please amend your detailed Financial Disclosure statement. This is published with the article. It must therefore be completed in full sentences and contain the exact wording you wish to be published.
1) State the initials, alongside each funding source, of each author to receive each grant. For example: "This work was supported by the National Institutes of Health (####### to AM; ###### to CJ) and the National Science Foundation (###### to AM)."
2) State what role the funders took in the study. If the funders had no role in your study, please state: "The funders had no role in study design, data collection and analysis, decision to publish, or preparation of the manuscript."
3) If any authors received a salary from any of your funders, please state which authors and which funders.
A: The Financial Disclosure statement was amended as suggested.

**Reviewers' Comments:** Reviewer's Responses to Questions

**Part I – Summary** Please use this section to discuss strengths/weaknesses of study, novelty/significance, general execution and scholarship.

Reviewer #1: In the manuscript, *Acinetobacter baumannii* lipooligosaccharide core region enhances CD14-dependent TLR4 endocytosis and boosts virulence through interferon-β production, Lee and Chen et al. demonstrate that the core region of *A. baumannii* LOS stimulates an immune response. This finding negates previous dogma that only Lipid A is the stimuli required for LPS/LOS derived inflammation. As such, I believe this work should be published in a high-tier journal such as PLoS Pathogens. I only request that the questions below be addressed:

Reviewer #2: In this work, Yi-Tzu Lee et al. investigate the role of the core region of *A. baumannii* LOS in host immune response. They use membrane vesicles from the different *A. baumannii* strains (WT, ∆lpsB and complemented strain) to show that the presence of a full core LOS binds better to CD14 and leads to CD14-dependent TLR4 endocytosis. Finally, the authors show that increased survival of mice challenged with *A. baumannii* membrane vesicles correlates with decreased levels of interferon-B production proposing a central role of LOS core in *A. baumannii* virulence.

Reviewer #3: This manuscript dissects the effect of the *A. baumannii* LOS core region on different types of TLR4 signaling. Specifically, using a lpsB mutant defective in producing the LOS core, data show that that MyD88-depedent signaling is unaffected while CD14 binding and CD14-dependent signaling is reduced, suggesting that the *A. baumannii* LOS core promotes IFN-B production and virulence. These data are overall strong and convincing.

It seems that the major finding is that in addition to CD14 being required for recognizing smooth LPS from e.g. *E. coli*, CD14 is also able to bind and differentiate between A. baumannii intact LOS (with a much shorter polysaccharide region) and re-LOS with only KDO on lipid A. A schematic figure may help clarify the major finding of the study.
In summary, this manuscript elucidates a role for CD14-dependent TLR4 discrimination between intact *A. baumannii* LOS with core saccharides and KDO-lipid A, further expanding our understanding of TLR4 signaling with implications for numerous gram-negative pathogens that encode LOS. However, the specific link between LOS core, CD14-dependent signaling, IFN-B production and virulence are not directly tested.

**Part II – Major Issues: Key Experiments Required for Acceptance**

Reviewer #1: Lines 49-50 – Have the role of MVs for *A. baumannii* been shown *in vivo*?

A: Thank you for your careful review. The role of MVs of *A. baumannii* have been shown *in vivo*. As described in Line 50-51, MVs released from the strains are collected and used for animal studies. We demonstrated that MV containing LOS exhibit higher pathogenicity in mice. Furthermore, as noted in Lines 53-54, the enhancement of interferon-β production was correlated with increased pathogenicity in the animal model.

Line 130-132 – While MVs have the known differences in core oligosaccharide content, it is unclear if differences in protein content could lead to a potential phenotype.

A: Although we did not analyze the protein content of MVs containing LOS and Re-LOS, it is unlikely that protein components contributed to the observed differences in IFN-β secretion. Treatment with proteinase K did not affect IFN-β secretion, whereas treatment with polymyxin B completely abolished it (Fig 2b). These results indicate that LOS and Re-LOS are the primary contributors to IFN-β induction.

Lines 339-350 – It is unclear how MVs are quantified. Given the different amounts of LPS, there could be different amounts of MVs produced, skewing possible results. If MVs were not quantified microscopically, results could be questionable due to inconsistent dosage.

A: Thank you for your careful review. MVs were quantified using a nanoparticle tracking analyzer (ZetaView PMX120). For all experiments involving MV challenges, the MVs were normalized based on particle number. These details have been added to the main text and the Materials and Methods section to avoid any confusion.

Line 650 – 40 ug of MVs is questionable when content of the MVs could greatly differ between WT and mutant bacteria, despite being relatively similar in size.

A: Thank you for your kind review. We repeated the animal experiments using MVs quantified based on particle number. Please find the revised manuscript attached.

Reviewer #2: - Growth of the wildtype, ∆lpsB and complemented strains in the conditions of infections should be added to the supplementary as difference in LOS could lead to decrease fitness, impacting the interpretation of experiments in Figure 1.

A: The colony-forming units (CFUs) of the three strains were measured at 4 and 24 h post-infection. At 24 h, the *∆lpsB* strain showed a slightly lower CFU compared to the wild-type and complemented strains (Fig 1e in the manuscript), suggesting a potential compromise in the fitness of the *∆lpsB* strain. However, no significant differences in CFU were observed among the three strains at 4 h post-infection, a time point at which pathogenic changes had already occurred. This indicates that the observed pathogenic effects were not attributable to differences in bacterial burden. To eliminate confounding effects related to bacterial fitness, we conducted subsequent experiments using MVs. The CFU data at 4 and 24 h post-infection are presented in Figure 1e.

- Although the pull down gives a qualitative result on the binding of CD14 to LOS, the authors should use an additional more quantitative method to determine the affinity of CD14 to the different LOS.

A: Further optimization of the experimental conditions is required; therefore, no additional data on the affinity of CD14 for the different LOS variants are currently available. Accordingly, we have softened the wording related to CD14’s affinity for the different LOS in the revised manuscript.

- A lot of details on experimental procedures including bacterial inoculum (CFU), reagents and inhibitors concentration, as well as complete procedures are missing from the material and method, complicating the understanding of some procedures.

A: Thank you for your reminder. To keep the manuscript concise, some details in the Materials and Methods section were omitted in the previous submission. In this revised version, we have added comprehensive information on the experimental procedures.

- The text should be edited to ensure language as well as scientific clarity.

A: Thank you for your suggestion regarding language and clarity. We have carefully re-reviewed and further refined the manuscript’s language, as well as reorganized the flow of the figures to improve overall clarity and readability. Please find the editing certificate attached at the end of the response letter.

Reviewer #3: 1. Experimental details with membrane vesicles are not clear, e.g. mouse experiment in Figure 1, Figure 2, Figure 3, . It is not clear how the MV were quantified and normalized for the mouse experiment. The inocula should be normalized for appropriate interpretation. Related, Table S1 shows the lpsB mutant has increased size of MV—how was this measured? If it was by microscopy, please display the images.

A: The size distribution and concentration of MVs were analyzed using a ZetaView PMX120 nanoparticle tracking analyzer (Particle Metrix GmbH, Germany). For subsequent challenge experiments, MVs were normalized based on particle number. Representative images of the MVs are provided below.

Ab908 MV

Ab908*∆lpsB* MV

Ab908*∆lpsB::lpsB* MV

2. Some additional controls are needed: In Figure 4e, the GAPDH loading controls must be shown for the 1 h samples and the 6 h samples. For all experiments using inhibitors, controls showing maintenance of viability or secretion of other cytokines/chemokines are needed. E.g. Fig 3b, Fig 3c, Fig 4f, Fig 4i, Fig. 5a.

A: Thank you for your reminder. GAPDH loading controls for both the 1-hour and 6-hour time points have been included in the revised Figure 4e. The cytotoxicity of the inhibitors was assessed using the CCK assay and is presented in S3 Fig. Cell viability was maintained under all inhibitor treatment conditions.

3. The link between LOS, IFN-B production, and virulence, remains inferred rather than directly interrogated. This should be addressed in the title and results. For Figure 6, the results section over concludes the linking of LOS core-dependent IFN-b signaling to *A. baumannii* virulence, “These results indicated that the LOS-enhanced IFN-β production was associated with the increased virulence of *A. baumannii* in the mice.” To test that, IFN-b and/or IFNAR-/- mice could be combined with *A. baumannii* lpsB mutants: if the model is correct, there should be no difference in virulence between MV from WT v. lpsB in IFNAR-/- or anti-IFN-b-treated mice. Additionally, it is not clear how the fact that IFNAR-/- also blocking IFN-a production would obscure a role for IFNAR in LOS- and CD14-dependent IFN-b production being important. Finally, for Figure 6b, the appropriate control is isotype antibody rather than untreated.

A: Thank you for your comments. We have repeated the experiments, with MVs normalized based on particle number. First, we determined the appropriate MV dose that induced lethality in wild-type mice (Fig 1j). Wild-type and IFNAR1-deficient mice were then challenged with MVs containing either intact LOS or LOS lacking the core region (Re-LOS) (Fig 6a). For the experiment shown in Figure 6b, an isotype control antibody was used for comparison.

The results demonstrated that IFNAR1-deficient mice exhibited a higher survival rate than wild-type mice when challenged with MVs containing intact LOS (Fig 6a). Furthermore, treatment with an anti-IFN-β antibody significantly improved survival in a dose-dependent manner compared to treatment with the isotype control (Fig 6b). These findings indicate that IFN-β production induced by LOS contributes to the increased pathogenicity of *A. baumannii* in mice.

However, not all IFNAR1-deficient mice survived following challenge with MVs containing intact LOS, and their survival rate remained lower than that of mice challenged with Re-LOS-containing MVs. This suggests that, beyond IFN-β signaling, intact LOS may contribute to increased pathogenicity through additional mechanisms. Further studies—such as those employing IFNAR1-deficient cells—are warranted to elucidate other potential mechanisms by which the LOS core region contributes to enhanced pathogenicity.

These data and interpretations have been incorporated into the revised manuscript.

**Part III – Minor Issues: Editorial and Data Presentation Modifications**
Please use this section for editorial suggestions as well as relatively minor modifications of existing data that would enhance clarity.

Reviewer #1:

Fig. 1 – Bacteria in figures do not resemble *A. baumannii* (flagella should not be present).

A: Thank you for your thoughtful review. We’ve redrawn the figure and remove the flagella of the bacteria.

Fig. 1C – Unclear if statistical testing is between WT vs. mutant, or Complement vs. Mutant.

A: Thank you for your reminder. The statistical testing was performed between WT vs. mutant. We’ve added the description for the revised figure legend.

Line 124 – It is unclear why bacterial burdens would be tested at 4 h when burdens would most likely be different later during infection.

A: We assessed bacterial burden at 4 and 24 h post-infection. The data for the 24-h time point are presented in Fig 1e. At 24 h post-infection, the bacterial burden of the strain containing Re-LOS was slightly lower compared to the other strains. However, at 4 h post-infection, bacterial burdens were similar across all strains, despite the observation of early pathogenic changes during this period. Since the pathogenic effects observed at 24 h could be influenced by differences in bacterial burden—and our aim was to specifically investigate the role of the LOS core independent of bacterial load—we selected the 4-h time point for further analysis. Additionally, to eliminate confounding effects related to bacterial viability and burden, we used MVs in the subsequent experiments.

Lines 127-129 – Why are IL-1B, IL-6, IL-10, and KC ignored in the results section, when clearly something strange appears to be happening with these levels?

A: We appreciate the reviewer’s comment. Several studies have reported that cytokine levels in BALF and serum may not consistently increase in patients with pneumonia (Eur Respir J. 1996; 9(9):1858–67. doi: 10.1183/09031936.96.09091858; BMC Pulm Med. 2004; 4:6. doi: 10.1186/1471-2466-4-6). Moreover, discrepancies between BALF and serum cytokine concentrations may reflect differences in the extent of local airway versus systemic inflammation (Front Immunol. 2022; 12:760488. doi: 10.3389/fimmu.2021.760488).

Given that TNF-α and IFN-β are among the primary cytokines induced in response to LPS, we focused our investigation on these specific cytokines.

Supplementary Fig. S2b – Infb1 is not highlighted in the volcano plot.

A: Thank you for your thoughtful comment. We have now added Ifnb1 and highlighted it in S2b Fig.

Line 150-152 – This is not a complete sentence.

A: Thank you for your reminder. We have made the correction in the revised manuscript. The sentence now reads as follows:

"Gene Set Enrichment Analysis (GSEA) revealed that the top five upregulated biological processes were 'response to IFN-β', 'cellular response to IFN-β', 'negative regulation of type I interferon-mediated signaling pathway', 'adhesion of symbiont to host', and 'regulation of response to IFN-γ', as shown in Figure 2e."

Fig. 2F – One of the WT heat maps looks completely different from the other 2 replicates. Can the authors explain this?

A: Thank you for your comment. We appreciate the reviewer’s careful observation. Indeed, replicate Ab908_2 exhibited a gene expression profile that diverged somewhat from the other two WT replicates. All RNA-seq samples were obtained from three independent biological replicates, and such variation may occur due to inherent biological variability.

Importantly, our key conclusions are supported by multiple independent lines of evidence. As shown in Fig 2a–2d and Fig 3a–d, MVs containing intact LOS consistently induced higher IFN-β secretion and gene expression—as well as enhanced signaling—compared to MVs with Re-LOS. Furthermore, Fig 4c–4f demonstrate that MVs stimulate IFN-β release via the IRF3 pathway, which is consistent with the transcription factor binding motif enrichment observed in S2 Table.

Taken together, these complementary datasets reinforce the overall RNA-seq findings. While Ab908_2 shows some divergence, it does not compromise the validity of the main conclusions.

Lines 213-216 – The inhibitors used are not readily known by some readers, so more explanation may be necessary.

A: Thank you for your reminder. We’ve added more explanation of the inhibitors in the revised manuscript.

Line 320 – Can the sources, isolation site, isolation year, and the “many antimicrobials” resistant to be noted? This could be of great value to the field.

A: Thank you for your comments. The detailed information of the clinical isolates was added in the S3 Table in the revised manuscript.

Lines 340-345. – Centrifugation speed should always be given in x g, as RPM varies by centrifuge.

A: Thank you for your reminder. Centrifugation speed were given in x g as suggested.

Line 602 – Does MOI refer to 500 MVs/cell or 500 cells producing MVs/cell

A: MOI refers to 500 MVs/cell. We’ve added the description in the revised manuscript.

Reviewer #2: - Following experiments in mice, the authors choose to investigate the response of macrophages, and they then alternate between experiments in THP-1 vs J774.a1 cells, it is unclear whether there is a rational for the use of one or the other cell type.

A: Thank you for your thoughtful comment. In this study, we initially observed cytokine alterations in mice. To assess whether these findings were specific to murine models or more broadly applicable across species, we conducted in vitro experiments using both murine (J774.A1) and human (THP-1) macrophage cell lines. The consistent responses observed in both cell types suggest a conserved mechanism.

For subsequent mechanistic studies, particularly those involving the IFN-β signaling pathway, we utilized a commercially available THP-1 reporter cell line. This cell line provides a robust and convenient system for assessing pathway activation, thereby facilitating more detailed investigation.

- Some figure sub-panels are not discussed in the text.

A: Thank you for your comments. We’ve added the discussion for the figure sub-panels in the revised manuscript.

- The authors do not discuss the inconsistency of other cytokines in between the different experiments. Could the authors speculate on these differences?
A: Thank you for your insightful comment. Several studies have reported that cytokine levels in bronchoalveolar lavage fluid (BALF) and serum may not consistently elevate in patients with pneumonia (Eur Respir J. 1996;9(9):1858-67. doi: 10.1183/09031936.96.09091858; BMC Pulm Med. 2004;4:6. doi: 10.1186/1471-2466-4-6). Furthermore, discrepancies between BALF and serum cytokine concentrations likely reflect differences in the extent of local airway inflammation versus systemic inflammation (Front Immunol. 2022;12:760488. doi: 10.3389/fimmu.2021.760488).

Given that TNF-α and IFN-β are the primary cytokines produced in response to LPS stimulation, our study focused mainly on these two cytokines.

- Figure legends should include the description of abbreviations used in the figures.

A: Thank you for your kind review. We’ve added the abbreviations used in the figures in the revised manuscript.

- Kinetics of killing using IP membrane vesicle appears rather inconsistent between experiments, how do the authors choose/normalize the number of vesicles injected?
Did the authors verify that the MV had a similar protein content? What quantity of MV is released by a certain number of bacteria?

A: Thank you for your valuable comments. We have repeated the animal studies using MVs normalized by particle number for the challenge, which has resulted in more consistent kinetics of mouse survival across experiments.

While we did not perform a direct comparison of protein content between different MVs, our data indicate that MVs containing proteins play little, if any, role in modulating IFN-β production. This conclusion is supported by the observation that treatment with proteinase K did not affect the pattern of IFN-β secretion, whereas treatment with polymyxin B completely abolished IFN-β production.

The amounts of MVs released by the bacteria was around 0.04-0.08 particle/CFU. There was no significant difference among three isolates.

- The authors should increase the size of the Figure 1a.

A: Thank you for your kind review. We’ve increased the size of the Figure 1a

- Figure S1c- according to the literature, the authors should be able to detect the LOS without inner/outer core generated in ∆lpsB mutant – what are the higher molecular weight bands?

A: Thank you for your insightful comment. The Re-LOS produced by the *∆lpsB* mutant is likely too small to be retained under our gel electrophoresis conditions. According to a previous study, the LOS from *Acinetobacter baumannii* is approximately 7 kDa (Infect Immun. 2010 Mar 1;78(5):2017–2023. doi: 10.1128/IAI.00016-10), and the Re-LOS is expected to be even smaller, which likely accounts for its absence in the detected bands. Regarding the higher molecular weight bands observed (16–27 kDa), their identity remains uncertain, but they are most likely non-specific protein bands rather than LOS species.

- Figure 2f – The color scale is missing.

A: Thank you for your kind review. We’ve added the color scale in the revised manuscript.

- Line 147 - The authors should describe the RNA-seq experiment in the text as well as which cells were used.

A: Thank you for your kind review. We have added the description in the revised manuscript.

- Line 152 – It appears that a piece of a sentence is missing
A: Thank you for your reminder. We have modified that in the revised manuscript. The sentence should be as the follows: Gene Set Enrichment Analysis (GSEA) revealed that the top five upregulated biological processes were “response to IFN-β”, “cellular response to IFN-β”, “negative regulation of type I interferon-mediated signaling pathway”, “adhesion of symbiont to host”, and “regulation of response to IFN-γ” as shown in Fig 2e.

- Line 152-155. It is unclear in the text why the authors only describe Irf1, Infb1 and Stat2.

A: Thank you for your comments. In our manuscript, we highlighted Irf1, Infb1, and Stat2 because these three genes showed significant and representative changes that directly reflect the activation of type I interferon signaling pathways, particularly in response to intact LOS-containing MVs. Specifically:

(a) Infb1 is the central gene encoding IFN-β, which is the key cytokine induced by *A. baumannii*-derived MVs and is the main focus of our study (Fig 2, 3, 4, 6 and S2b Fig).
(b) Irf1 is a transcription factor that is downstream of IFN-β signaling and was significantly upregulated in the “response to IFN-β” and “cellular response to IFN-β” gene ontology terms, according to our heatmap of GSEA and volcano plots of differential expression genes results (Fig 2e & S2b Fig).
(c) Stat2 was notably upregulated in the category of “negative regulation of type I interferon-mediated signaling pathway,” indicating its potential role in feedback modulation of IFN-β signaling (Fig 2e & S2c Fig).

While other genes were differentially expressed (as shown in S2 Fig), we selected these three as representative markers to illustrate distinct aspects of IFN-β production, IFN-mediated downstream signaling, and regulatory feedback, respectively. These genes served as focal points to support the functional relevance of the TRIF-dependent TLR4 pathway activated by the core region of LOS.

- A few references are missing in the results section.

A: Thank you for your kind review. We’ve added references in the revised manuscript.

Reviewer #3: 1. In the opening sentence of the abstract, virulence typically refers to ability to cause infection by the organism, not molecules such as lipooligosaccharides, so please reword.
A: Thank you for your suggestion. We’ve replaced “virulence” with “pathogenesis” in the revised manuscript.

2. The manuscript would benefit from additional copyediting throughout.
A: Thank you for your suggestion regarding language and clarity. We have carefully re-reviewed and further refined the manuscript’s language, as well as reorganized the flow of the figures to improve overall clarity and readability. Please find the editing certificate attached at the end of the response letter.

3. Lines 113-116 appear to be out of place and include figure callouts out of order.
A: Thank you for your comments. We’ve revised the manuscript accordingly.

4. Figure 1 A some of the text is too small to read.

A: Thank you for your kind review. We’ve increased the size of the Fig 1a.

5. Lines 127-128: Most of the cytokine results are not discussed. It seems odd that the patterns would be so different for some between serum and BALF (e.g. IL-6, KC)—what might these mean for understanding inflammation induced by intact LOS?

A: We appreciate the reviewer’s comment. Several literatures have documented that BALF and serum cytokines may not consistently elevate in patients with pneumonia.(Eur Respir J. 1996;9(9):1858-67. doi: 10.1183/09031936.96.09091858.; BMC Pulm Med. 2004;4:6. doi: 10.1186/1471-2466-4-6.) Meanwhile, the discrepancy between BALF and serum concentration could be a result from the different extent of local inflammatory airway and systemic inflammation.(Front Immunol. 2022;12:760488. doi: 10.3389/fimmu.2021.760488.) While TNF-α and IFN-β are the main cytokines produced in response to LPS, we focused on these cytokines in our research.

6. Why is MIP-2 (murine inflammatory protein 2) shown for Figure 2A with human THP1 macrophages?
A: Thank you for your kind review. The MIP-2 should be “CXCL2”. We’ve removed cytokines other than IFN-β and TNF-α in the revised manuscript.

7. What are the sequence types of strains shown in Figure 2D? A: The sequence types of the strains shown in Fig 2d have been listed in the Table S3. The Ab908, Ab1033, and Ab2804 belong to ST208, ST218, and ST473, respectively. We’ve also added the information in the revised manuscript to make it more readable.

8. Figure 5: typo-picentannol should be piceatannol
A: Thank you for your kind review. We’ve corrected the typo in the revised manuscript.

9. Figure 5e: was the difference in pulldown of LOS by CD14 statistically significant?

A: Thank you for your thoughtful review. While the data is so limited, there was no significant difference in pulldown between the LOS of different isolates.

**Editing Certificate**
