# Supplementary material for: Acinetobacter baumannii lipooligosaccharide core region promotes CD14-dependent TLR4 endocytosis and enhances pathogenicity through interferon-β production
Source: PLoS Pathog. 2026 Jul 14;22(7):e1014364. doi: 10.1371/journal.ppat.1014364 (PMC13367702; doi:10.1371/journal.ppat.1014364)
Supplement: Attachment — Submitted filename: Response to Reviewers 0311.docx [file ppat.1014364.s010.docx]

**Response letter**

**Manuscript ID: PPATHOGENS-D-25-00850R1
*Acinetobacter baumannii* lipooligosaccharide core region enhances CD14-dependent TLR4 endocytosis and boosts virulence through interferon-β production**

Dear Editor and Reviewers,

We sincerely thank you for your careful re-evaluation of our manuscript and for the constructive and insightful comments. We have thoroughly addressed all concerns and performed additional experiments to strengthen the mechanistic conclusions of our study. The major revisions include:

1. Repetition of the animal experiment (Fig. 1J) using membrane vesicles (MVs) quantified based on particle number.
2. Repetition of LOS extraction and silver staining to confirm truncated LOS in the *ΔlpsB* mutant.
3. PCR mapping to confirm double-crossover deletion of the *lpsB* gene.
4. Restoration and clarification of the cytokine response data.

Below, we provide point-by-point responses.

Thank you once again for your time and consideration.

Warm regards,

Yung-Chih Wang

Division of Infectious Disease and Tropical Medicine, Department of Internal Medicine, Tri-Service General Hospital, National Defense Medical University

7F, No. 325, Section 2, Cheng-Kung Road, Neihu 114, Taipei, Taiwan

Tel: +886-2-87927257

Fax: +886-2-87927258

Email: [wystwyst@gmail.com](mailto:wystwyst@gmail.com)

**Additional Editor Comments :**

Two of the original reviewers have re-evaluated this manuscript and remain concerned about several experiments that must be fully addressed in a potential revision. The revised version should experimentally resolve the issue regarding the LOS pattern, as raised by both reviewers. One reviewer noted that “the pulldowns do not appear to show higher affinity for intact LOS to CD14 compared to LBP, as LBP shows ~40 LOS/protein while CD14 shows ~30 LOS/protein.” In the revised version, I expect the authors to provide quantitative measurements of affinity. The issue regarding cytokine responses should also be clarified. If the authors convincingly address these and the additional concerns stated by both reviewers, I would be willing to consider a revised version.
**A:** Thank you for these important comments.

(1) We have repeated the LOS extraction and silver staining experiments and now provide clear confirmation of *lpsB* deletion and complementation in the revised manuscript.

(2) At present, we are unable to obtain sufficiently purified LOS to perform Surface Plasmon Resonance (SPR) analysis for quantitative affinity measurement. We are continuing efforts to optimize LOS purification. Given this limitation, we have removed the binding affinity results and related discussion to avoid overstating our conclusions.

(3) We have also restored and clarified the cytokine response data in the revised manuscript.

**Journal Requirements:**

1) Regarding Figure 7, thank you for stating " We've redrawn the figure using the open source materials and uploaded the new Fig 7". Please include in the figure legend a link to the source of the images.

**A:** We have revised the Figure 7 legend to include the statement: “Created with BioArt Source and Wikimedia Commons.”

**Reviewers' Comments:**

Reviewer's Responses to Questions

**Part I - Summary**
Please use this section to discuss strengths/weaknesses of study, novelty/significance, general execution and scholarship.

Reviewer #2: (No Response)

Reviewer #3: The authors have addressed some of my previous comments. However, additional information they provide raises new questions on methods and some key experimental comments were not addressed.

**Part II – Major Issues: Key Experiments Required for Acceptance**

**Reviewer #2:**

A: The data obtained from the mouse experiments (Fig. 1J) appear to be the same as in the original manuscript, except normalized to vesicle number rather than 50 µg of protein. Without repeating the mice experiments, the authors should provide biological replicates demonstrating equivalence between the 50 µg protein and the vesicle number normalization, especially since vesicles from the *ΔlpsB* strain are larger.

**A:** Thank you for this important comment. We have repeated the animal experiments using MVs quantified based on particle number. The results are consistent with our previous findings. The updated data are presented in the revised Fig. 1J.

Q: The authors should repeat and include a LOS silver stain to clearly show the truncated LOS used in this study. Currently, it appears as if no LOS is present in the ΔlpsB strain. For reference, see PMID: 38349180 (Fig 7A), where LOS was extracted from ΔlpsB mutant and displayed a shorter LOS band.

**A:** We have repeated the LOS extraction and now include clear silver-stained gels in Supporting Information S1d Fig. The *ΔlpsB* mutant exhibits a truncated LOS pattern consistent with previous reports.

**Reviewer #3:**

Q1. Strain construction and Table S3-4:
a. First, ∆lpsB mutants are made in multiple strain backgrounds but only one set of primers is included. Are the sequences where the primers bind actually identical in all three strains? That seems unlikely.

**A:** The *lpsB* sequences in the three strains (Ab908, Ab1033, and Ab2388) share high sequence identity (~97.28%) as shown below. The primers used for knockout *lpsB* was presented in S4 Table and S1c Fig. The primer locations are now clearly indicated in the revised manuscript (S4 Table).

**S4 Table. Primers used for knockout and confirmation of the *lpsB* gene.**

| **Primers** | | **Sequences** | **Location or Target gene** | | |
| --- | --- | --- | --- | --- | --- |
| P1 | 5’-GTCTACACGAACCCTTTGGCAA-3’ | | | *Apra^R^* |  |
| P2 | 5’-TGGAACTTATGAGCTCAGCCAA-3’ | | | *Apra^R^* |  |
| P3 | 5’-**ATGAAAGTGATGCAACTTCT-**3’ | | | Upstream of *lpsB* |  |
| P4 | 5’-GGTTCGTGTAGAC**GACTTGGTTAAACCACTGT**-3’ | | | Upstream of *lpsB* |  |
| P5 | 5’-GCTCATAAGTTCCA**ACTTTGCCTACCT**-3’ | | | Downstream of *lpsB* |  |
| P6 | 5’-**TCAATTCAATACACTTTGAT**-3’ | | | Downstream of *lpsB* |  |
| P7 | 5’-AACGCAACAGCGACATCCATGT-3’ | | | Flanking of *lpsB* |  |
| P8 | 5’-GTAGGTAACGCGCTTGCTGTACTT-3’ | | | Flanking of *lpsB* |  |
| P9 | 5’-TGTAAAACGACGGCCAGT-3’ | | | pGEM T vector M13 forward sequencing primer binding site |  |
| P10 | 5’-CGTATATCTGAGCGATGTC-3’ | | | Downstream of *lpsB* |  |

^#^ The sequences shown in bold represent the sequence of *lpsB* gene.

**Sequence alignment of the *lpsB* of Ab908, Ab1033, and Ab2804**. The primers location was indicated.

dfvsvseve

P3→

←P4

P5→

←P6

b. Second, the description of the methods would not be likely to generate deletion mutants and there are no methods described or primers listed to verify the deletion mutants. Specifically, using 500 bp homology on either side is low efficiency, when combined with selecting for a double crossover in 1 step, it is extremely unlikely to select correct mutants. Second, there are no primers listed that anneal near the lpsB gene but outside of the construct present in the pGEM-lpsB-apraR that would be required to verify the mutant. Thus, the strains used may be single crossovers integrating pGEM-lpsB-apraR into the chromosome.

**(b)** The *ΔlpsB* mutants were generated via double-crossover homologous recombination. To confirm correct deletion, we performed PCR using a forward primer located upstream of *lpsB* and a reverse primer within the apramycin resistance cassette. Successful amplification (S1c Fig, left panel) confirmed replacement of *lpsB* with the apramycin cassette through double-crossover recombination. The primers used were provided in Table S4. The positions of the primers and the PCR fragments amplified using the indicated primer pairs are shown as S1c Fig. The plasmid pGEM-*lpsB::Apra* was included in the right panel as a positive control.

Q2. In Figure S1C, there should be detectable LOS without core sugars in an LOS gel, as previously reported (Hood et al., Infect. Immun., 2013). This comment from reviewer 2 remains a concern.

**A:** We have repeated LOS extraction and now include improved silver-stained gels in Supporting Information S1d Fig. The *lpsB* knockout mutant shows a truncated LOS band consistent with previously published findings.

Q3. It does not seem appropriate to remove all data for other cytokines as the response to reviewers querying about why the data for other cytokines appearing inconsistent.

**A:** We agree. We have restored the full cytokine panel (Fig. 2d) and provide an expanded discussion of these results in the revised manuscript.

Q4. Line 326-327/Figure 5e: the pulldowns do not appear to show higher affinity for intact LOS to CD14 compared to LBP, as the LBP shows ~40 LOS/protein while CD14 shows ~30 LOS/protein. In addition, as reviewers previously raised, this measure of binding is qualitative and additional methods/analyses should be used to measure affinity quantitatively to support the claims of the manuscript.

**A:** We acknowledge this concern. Due to limitations in obtaining highly purified LOS suitable for SPR analysis, we are currently unable to provide quantitative affinity measurements. To avoid overinterpretation, we have removed statements regarding differential binding affinity between CD14 and LOS or Re-LOS. Importantly, our central finding—that the LOS core region biases signaling toward TRIF-dependent pathways and enhances IFN-β production—remains well supported by the functional data presented.

**Part III – Minor Issues: Editorial and Data Presentation Modifications**
Please use this section for editorial suggestions as well as relatively minor modifications of existing data that would enhance clarity.

**Reviewer #2:**

Q: Line 37–38: The current sentence, “Lipooligosaccharides (LOS) with a core region are more pathogenic than those without it; however, the underlying mechanism remains fully elucidated,” is inaccurate, as an LOS molecule itself cannot be described as pathogenic. The sentence should be rephrased in line with line 68–69 which states: “An *A. baumannii* strain harboring the core region of LOS displayed higher virulence than a strain lacking this core region.” In addition, the phrase “remains fully elucidated” should be corrected to “remains to be fully elucidated.”

**A:** The sentence has been revised to accurately describe strain virulence rather than attributing pathogenicity directly to LOS. The phrase has also been corrected to “remains to be fully elucidated.”

Q: Some figure panels remain too small (e.g., 1B, 1C, 1J), and the labels/writing are difficult to read (e.g., 1D–H).

**A:** All figures have been resized and reformatted to improve clarity and readability.

Q: *Acinetobacter baumannii* is not curved, schematics in Fig. 1A, 1I, and S1B should be corrected.

**A:** The bacterial illustrations in Fig. 1A, 1I, and S1b have been corrected to reflect the proper morphology of *A. baumannii*.

- Fig. 2B: The label “to 1000 ng of LPS” on the y-axis is unclear and should be clarified.

**A:** The incorrect y-axis label has been removed.

Fig. 2F: The authors should specify what the scale numbers represent log₂(FC) or FC).

**A:** The scale represents log₂ fold change (log₂FC). This is now specified in the figure legend.

Q: Line 181: Please specify the strain/cell type used for RNA-seq.

**A:** RNA-seq experiments were performed using J774A.1 cells. This information has been added to the Materials and Methods and clarified in the Results section (Lines 182-183).

- Mice were injected with 8.3 × 10¹¹ particles. Given that each bacterium produces ~0.08 vesicles, this corresponds to ~1 × 10¹³ bacteria. Could the authors speculate whether these difference might still be relevant in an infection?

**A:** We agree that the administered particle number corresponds to a comparatively high bacterial load. This may be attributable to the relative resistance of C57BL/6 mice to *A. baumannii* infection and highlights an inherent limitation of this animal infection model.

- Fig. 4F: There appear to be issues with the statistical error bars that should be addressed

**A:** The statistical error bars have been corrected.

**Reviewer #3:**

Q5. The text in most of the figures is too small.

**A:** Thank you for your thoughtful review. We have resized and modified the figures in the revised manuscript.

6. The primers to generate complementation vectors are not reported nor any information about pAbYm2 such as antibiotic resistance.

**A:** The shuttle vector pYMAb2 was previously constructed in our laboratory. It is derived from the *Escherichia coli* plasmid pET-28a and contains a kanamycin resistance determinant, along with an SphI/XbaI fragment containing replicons from *A. baumannii* ATCC19606 plasmid pMAC (reference 44). This information has been added to the revised manuscript.

7. Table S3 cites reference 48 for the double crossover method, which does not appear to be the correct reference.

**A:** We apologize for the typographical error. The double-crossover method is described in the Materials and Methods (references 42 and 43). Reference 48 has been removed from Table S3.

8. Antibiotic resistance should be listed/specified for strains used in Table S3 as requested by another reviewer.

**A:** Antibiotic resistance markers for all strains have now been specified in the revised Table S3.
