# Supplementary material for: Acinetobacter baumannii lipooligosaccharide core region promotes CD14-dependent TLR4 endocytosis and enhances pathogenicity through interferon-β production
Source: PLoS Pathog. 2026 Jul 14;22(7):e1014364. doi: 10.1371/journal.ppat.1014364 (PMC13367702; doi:10.1371/journal.ppat.1014364)
Supplement: Attachment — Submitted filename: Response to Reviewers 0509.docx [file ppat.1014364.s011.docx]

**Response letter**

**Manuscript ID: PPATHOGENS-D-25-00850R2
*Acinetobacter baumannii* lipooligosaccharide core region enhances CD14-dependent TLR4 endocytosis and boosts virulence through interferon-β production**

Dear Editor and Reviewers,

We sincerely thank you for your careful re-evaluation of our manuscript and for the constructive and insightful comments. We have thoroughly addressed all concerns and performed additional experiments to strengthen the mechanistic conclusions of our study. The major revisions include:

1. Repetition of the sliver-stained gel experiment (S1d Fig) and included LPS from *E. coli* as a reference to aid interpretation.
2. Resize the text in the Figures to make them more readable.
3. Uploaded all the supporting files as the Supporting Information.

Below, we provide point-by-point responses.

Thank you once again for your time and consideration.

Warm regards,

Yung-Chih Wang

Division of Infectious Diseases and Tropical Medicine, Department of Internal Medicine, Tri-Service General Hospital, National Defense Medical University

7F, No. 325, Section 2, Cheng-Kung Road, Neihu 114, Taipei, Taiwan

Tel: +886-2-87927257

Fax: +886-2-87927258

Email: [wystwyst@gmail.com](mailto:wystwyst@gmail.com)

**Additional Editor Comments:**
Overall, the reviewers are pleased with most of the edits and changes in this revised version. However, both reviewers raise concerns about the quality of the SDS-PAGE analysis of LOS (LPS). This issue is not merely cosmetic; the authors need to provide a high-quality silver-stained gel showing the LOS profiles of both the wild-type and mutant strains. One reviewer also suggested including LPS from an additional bacterial species as a reference to aid interpretation. If the authors can improve the SDS-PAGE analysis to the required standard and revise the manuscript in accordance with the reviewers’ comments, the work would be suitable for acceptance.
**A:** Thank you for the comment. We sincerely apologize for the previous mistake regarding the incorrect labeling of the LOS position markers in the figure. In the revised manuscript, we modified the silver staining procedure and incorporated two *Escherichia coli* reference strains as controls to improve the clarity and reliability of LOS profile comparisons. Specifically, LPS derived from *E. coli* BW25113 lacking the *waaL* gene is deficient in the O-antigen, whereas LPS from *E. coli* BW25113 lacking the *waaC* gene exhibits a truncated structure with loss of the outer core and portions of the inner core. The gel showed obvious loss of LOS of the mutant strains. Please find the S1d Fig in the revised manuscript and below.

**S1d Fig. Silver stain of the purified LOS shows that *A. baumannii* 908 (Ab908), the complementary strain (Ab908Δ*lpsB*::*lpsB*), and *E. coli* BW25113*ΔwaaL* (Ec*ΔrfaL*) produce LOS with core, but not the *lps*B deletion mutant strain (Ab908Δ*lpsB*) and *E. coli* BW25113*ΔwaaC* (Ec*ΔrfaC*).**

Reference: Yethon JA, Vinogradov E, Perry MB, Whitfield C. Mutation of the lipopolysaccharide core glycosyltransferase encoded by waaG destabilizes the outer membrane of *Escherichia coli* by interfering with core phosphorylation. J Bacteriol. 2000 Oct;182(19):5620-3. doi: 10.1128/JB.182.19.5620-5623.2000.

**Journal Requirements:**
If the reviewer comments include a recommendation to cite specific previously published works, please review and evaluate these publications to determine whether they are relevant and should be cited. There is no requirement to cite these works unless the editor has indicated otherwise.

1) We have noticed that you have cited Tables S1, and S2 in the manuscript file but there are no corresponding tables in the manuscript. Please amend your manuscript to include these tables noting that tables should not be uploaded as individual files.

**A:** Thank you for your kindly reminder. We have uploaded the corresponding files as the 'Supporting Information' in the revised manuscript.

2) We have noticed that you have a list of Supporting Information legends in your manuscript. However, there are no corresponding files uploaded to the submission. Please upload them as separate files with the item type 'Supporting Information'.
**A:** Thank you for your kindly reminder. We have uploaded the corresponding files as the 'Supporting Information' in the revised manuscript.

**Reviewers' Comments:**
Reviewer's Responses to Questions

**Part I - Summary**
Please use this section to discuss strengths/weaknesses of study, novelty/significance, general execution and scholarship.

Reviewer #2: (No Response)

Reviewer #3: Overall, the additional primer and strain construction information clarifies the methods used and mutant generated. However, the LOS gel remains confusing.

**Part II – Major Issues: Key Experiments Required for Acceptance**
Please use this section to detail the key new experiments or modifications of existing experiments that should be absolutely required to validate study conclusions.
Generally, there should be no more than 3 such required experiments or major modifications for a "Major Revision" recommendation. If more than 3 experiments are necessary to validate the study conclusions, then you are encouraged to recommend "Reject".

Reviewer #2: The authors have addressed most of my comments.
However, the LOS silver stain remains unsatisfactory, both in terms of quality and interpretation (banding pattern).

**A:** Thank you for the comment. We sincerely apologize for the previous mistake regarding the incorrect labeling of the LOS position markers in the figure. In the revised manuscript, we modified the silver staining procedure and incorporated two *Escherichia coli* reference strains as controls to improve the clarity and reliability of LOS profile comparisons. Specifically, LPS derived from *E. coli* BW25113 lacking the *waaL* gene is deficient in the O-antigen, whereas LPS from *E. coli* BW25113 lacking the *waaC* gene exhibits a truncated structure with loss of the outer core and portions of the inner core. The gel showed obvious loss of LOS of the mutant strains. Please find the S1d Fig in the revised manuscript and below.

**S1d Fig. Silver stain of the purified LOS shows that *A. baumannii* 908 (Ab908), the complementary strain (Ab908Δ*lpsB*::*lpsB*), and *E. coli* BW25113*ΔwaaL* (Ec*ΔrfaL*) produce LOS with core, but not the *lps*B deletion mutant strain (Ab908Δ*lpsB*) and *E. coli* BW25113*ΔwaaC* (Ec*ΔrfaC*).**

Reference: Yethon JA, Vinogradov E, Perry MB, Whitfield C. Mutation of the lipopolysaccharide core glycosyltransferase encoded by waaG destabilizes the outer membrane of *Escherichia coli* by interfering with core phosphorylation. J Bacteriol. 2000 Oct;182(19):5620-3. doi: 10.1128/JB.182.19.5620-5623.2000.

Reviewer #3: 1. The LOS gel remains confusing. An *A. baumannii* lpsB mutant has been reported multiple times by multiple groups to generate LOS band slightly lower than the full length LOS (PMID: 23230287; PMID: 32908144; PMID: 38349180). However, these were all with *A. baumannii* 17978 so it may be different for Ab908. LOS typically migrates more similarly to ~10 kDa molecular weight. The band labeled LOS in Figure S1D at the dye front is not likely LOS, although it is not clear since there were no controls included here for comparison to e.g. rough type E. coli that runs similarly as *A. baumannii* LOS.

**A:** Thank you for the comment. We sincerely apologize for the previous mistake regarding the incorrect labeling of the LOS position markers in the figure. In the revised manuscript, we modified the silver staining procedure and incorporated two *Escherichia coli* reference strains as controls to improve the clarity and reliability of LOS profile comparisons. Specifically, LPS derived from *E. coli* BW25113 lacking the *waaL* gene is deficient in the O-antigen, whereas LPS from *E. coli* BW25113 lacking the *waaC* gene exhibits a truncated structure with loss of the outer core and portions of the inner core. The gel showed obvious loss of LOS of the mutant strains. Please find the S1d Fig in the revised manuscript.

**Part III – Minor Issues: Editorial and Data Presentation Modifications**
Please use this section for editorial suggestions as well as relatively minor modifications of existing data that would enhance clarity.

Reviewer #2: (No Response)

Reviewer #3: 1. Some text in figures remains too small, e.g. the axis labels in Figure 1

**A:** Thank you for your thoughtful review. We have resized and modified the Figures in the revised manuscript.

2. The *lpsB* deletion design appears to only delete 35 bp in the middle of the gene. This is fine as a partial deletion/insertion, but should likely be described as a disruption or knockout rather than deletion in the results. The description in the methods in Lines 465-468 is appropriate. However, Line 469-470 should be corrected, as the 500 bp homology arms appear to be within the *lpsB* gene rather than upstream and downstream of the gene.

**A:** Thank you for your thoughtful review. We have revised description to “Approximately 500 bp regions in R1 and R2, corresponding to the 5′-proximal and 3′-proximal regions of the *lpsB* coding sequence, were amplified using P3/P4 (R1 region) and P5/P6 (R2 region) primers.” in the revised manuscript.

3. It seems P8 is not depicted in the primer scheme in Figure S1C.

**A:** Thank you for your careful reminder. We have corrected that in the S1C fig in the revised manuscript as shown below.

**Figure resubmission:**

While revising your submission, we strongly recommend that you use PLOS’s NAAS tool (https://ngplosjournals.pagemajik.ai/artanalysis) to test your figure files. NAAS can convert your figure files to the TIFF file type and meet basic requirements (such as print size, resolution), or provide you with a report on issues that do not meet our requirements and that NAAS cannot fix.

After uploading your figures to PLOS’s NAAS tool - https://ngplosjournals.pagemajik.ai/artanalysis, NAAS will process the files provided and display the results in the "Uploaded Files" section of the page as the processing is complete. If the uploaded figures meet our requirements (or NAAS is able to fix the files to meet our requirements), the figure will be marked as "fixed" above. If NAAS is unable to fix the files, a red "failed" label will appear above. When NAAS has confirmed that the figure files meet our requirements, please download the file via the download option, and include these NAAS processed figure files when submitting your revised manuscript.
