# Supplementary material for: Acinetobacter baumannii lipooligosaccharide core region promotes CD14-dependent TLR4 endocytosis and enhances pathogenicity through interferon-β production
Source: PLoS Pathog. 2026 Jul 14;22(7):e1014364. doi: 10.1371/journal.ppat.1014364 (PMC13367702; doi:10.1371/journal.ppat.1014364)
Supplement: Peer review history

# Peer review: Decision Letter 0

**DOI:** 10.1371/journal.ppat.1014364.r001

**Authors:** Matthew Wolfgang, Mario Feldman

25 May 2025

-->PPATHOGENS-D-25-00850

Acinetobacter baumannii lipooligosaccharide core region enhances CD14-dependent TLR4 endocytosis and boosts virulence through interferon-β production

PLOS Pathogens

Dear Dr. Wang,

Thank you for submitting your manuscript to PLOS Pathogens. After careful consideration, we feel that it has merit but does not fully meet PLOS Pathogens's publication criteria as it currently stands. Therefore, we invite you to submit a revised version of the manuscript that addresses the points raised during the review process.

Please submit your revised manuscript within 60 days Jul 24 2025 11:59PM. If you will need more time than this to complete your revisions, please reply to this message or contact the journal office at plospathogens@plos.org. When you're ready to submit your revision, log on to https://www.editorialmanager.com/ppathogens/ and select the 'Submissions Needing Revision' folder to locate your manuscript file.

Please include the following items when submitting your revised manuscript:

* A rebuttal letter that responds to each point raised by the editor and reviewer(s). You should upload this letter as a separate file labeled 'Response to Reviewers'. This file does not need to include responses to any formatting updates and technical items listed in the 'Journal Requirements' section below.

* A marked-up copy of your manuscript that highlights changes made to the original version. You should upload this as a separate file labeled 'Revised Manuscript with Track Changes'.

* An unmarked version of your revised paper without tracked changes. You should upload this as a separate file labeled 'Manuscript'.

If you would like to make changes to your financial disclosure, competing interests statement, or data availability statement, please make these updates within the submission form at the time of resubmission. Guidelines for resubmitting your figure files are available below the reviewer comments at the end of this letter.

We look forward to receiving your revised manuscript.

Kind regards,

Mario F Feldman

Academic Editor

PLOS Pathogens

Matthew Wolfgang

Section Editor

PLOS Pathogens

Sumita Bhaduri-McIntosh

Editor-in-Chief

PLOS Pathogens

orcid.org/0000-0003-2946-9497

Michael Malim

Editor-in-Chief

PLOS Pathogens

orcid.org/0000-0002-7699-2064

**Additional Editor Comments:**

Three experts in the topic have revised this manuscript. The three of them found the work interesting. However, they have raised concerns that need to be addressed in order to fully support the authors' conclusions. In particular, the work with OMV needs more quantifications/controls. Visualization by microscopy and proper LOS/protein quantifications are needed. The affinity of CD14 to the different LOS needs to be investigated by other methods. In Figure 4e, the GAPDH loading controls must be shown for the 1 h samples and the 6 h samples. For all experiments using inhibitors, controls showing maintenance of viability or secretion of other cytokines/chemokines are needed. E.g. Fig 3b, Fig 3c, Fig 4f, Fig 4i, Fig. 5a. Finally, there are concerns about the results in Figure 6 that require more work to be conclusive. Details pm experimental procedures including bacterial inoculum (CFU), reagents and inhibitors concentration, as well as complete procedures are missing from Material and Methods.

**Journal Requirements:**

1) Please ensure that the CRediT author contributions listed for every co-author are completed accurately and in full.

At this stage, the following Authors/Authors require contributions: Yi-Tzu Lee, Te-Li Chen, Shu-Chen Kuo, Yu-Han Liu, Meng-Chang Lee, Ming-Fen Chuang, Mei-Chei Tan, I-Ming Lee, Ya-Sung Yang, and Yung-Chih Wang. Please ensure that the full contributions of each author are acknowledged in the "Add/Edit/Remove Authors" section of our submission form.

The list of CRediT author contributions may be found here: https://journals.plos.org/plospathogens/s/authorship#loc-author-contributions

2) We do not publish any copyright or trademark symbols that usually accompany proprietary names, eg ©,  ®, or TM  (e.g. next to drug or reagent names). Therefore please remove all instances of trademark/copyright symbols throughout the text, including:

- TM on pages: 32, 33, 34, and 35.

3) We have noticed that you have uploaded Supporting Information files, but you have not included a list of legends. Please add a full list of legends for your Supporting Information files after the references list.

4) Some material included in your submission may be copyrighted. According to PLOSu2019s copyright policy, authors who use figures or other material (e.g., graphics, clipart, maps) from another author or copyright holder must demonstrate or obtain permission to publish this material under the Creative Commons Attribution 4.0 International (CC BY 4.0) License used by PLOS journals. Please closely review the details of PLOSu2019s copyright requirements here: PLOS Licenses and Copyright. If you need to request permissions from a copyright holder, you may use PLOS's Copyright Content Permission form.

Please respond directly to this email and provide any known details concerning your material's license terms and permissions required for reuse, even if you have not yet obtained copyright permissions or are unsure of your material's copyright compatibility. Once you have responded and addressed all other outstanding technical requirements, you may resubmit your manuscript within Editorial Manager.

Potential Copyright Issues:

i) Figures 1A, 1I, 5E, and S1B. Please confirm whether you drew the images / clip-art within the figure panels by hand. If you did not draw the images, please provide (a) a link to the source of the images or icons and their license / terms of use; or (b) written permission from the copyright holder to publish the images or icons under our CC BY 4.0 license. Alternatively, you may replace the images with open source alternatives. See these open source resources you may use to replace images / clip-art:

- https://commons.wikimedia.org

- https://openclipart.org/.

5) Please amend your detailed Financial Disclosure statement. This is published with the article. It must therefore be completed in full sentences and contain the exact wording you wish to be published.

1) State the initials, alongside each funding source, of each author to receive each grant. For example: "This work was supported by the National Institutes of Health (####### to AM; ###### to CJ) and the National Science Foundation (###### to AM)."

2) State what role the funders took in the study. If the funders had no role in your study, please state: "The funders had no role in study design, data collection and analysis, decision to publish, or preparation of the manuscript."

3) If any authors received a salary from any of your funders, please state which authors and which funders..

**Reviewers' Comments:**

Reviewer's Responses to Questions

**Part I - Summary**

Please use this section to discuss strengths/weaknesses of study, novelty/significance, general execution and scholarship.

Reviewer #1: In the manuscript, Acinetobacter baumannii lipooligosaccharide core region enhances CD14-dependent TLR4 endocytosis and boosts virulence through interferon-β production, Lee and Chen et al. demonstrate that the core region of A. baumannii LOS stimulates an immune response. This finding negates previous dogma that only Lipid A is the stimuli required for LPS/LOS derived inflammation. As such, I believe this work should be published in a high-tier journal such as PLoS Pathogens. I only request that the questions below be addressed:

Reviewer #2: In this work, Yi-Tzu Lee et al. investigate the role of the core region of A. baumannii LOS in host immune response. They use membrane vesicles from the different A. baumannii strains (WT, ∆lpsB and complemented strain) to show that the presence of a full core LOS binds better to CD14 and leads to CD14-dependent TLR4 endocytosis. Finally, the authors show that increased survival of mice challenged with A. baumannii membrane vesicles correlates with decreased levels of interferon-B production proposing a central role of LOS core in A. baumannii virulence.

Reviewer #3: This manuscript dissects the effect of the A. baumannii LOS core region on different types of TLR4 signaling. Specifically, using an lpsB mutant defective in producing the LOS core, data show that that MyD88-depedent signaling is unaffected while CD14 binding and CD14-dependent signaling is reduced, suggesting that the A. baumannii LOS core promotes IFN-B production and virulence. These data are overall strong and convincing.

It seems that the major finding is that in addition to CD14 being required for recognizing smooth LPS from e.g. E. coli, CD14 is also able to bind and differentiate between A. baumannii intact LOS (with a much shorter polysaccharide region) and re-LOS with only KDO on lipid A. A schematic figure may help clarify the major finding of the study.

In summary, this manuscript elucidates a role for CD14-dependent TLR4 discrimination between intact A. baumannii LOS with core saccharides and KDO-lipid A, further expanding our understanding of TLR4 signaling with implications for numerous gram-negative pathogens that encode LOS. However, the specific link between LOS core, CD14-dependent signaling, IFN-B production and virulence are not directly tested.

**********

**Part II – Major Issues: Key Experiments Required for Acceptance**

Please use this section to detail the key new experiments or modifications of existing experiments that should be absolutely required to validate study conclusions.

Generally, there should be no more than 3 such required experiments or major modifications for a "Major Revision" recommendation. If more than 3 experiments are necessary to validate the study conclusions, then you are encouraged to recommend "Reject".

Reviewer #1: Lines 49-50 – Have the role of MVs for A. baumannii been shown in vivo?

Line 130-132 – While MVs have the known differences in core oligosaccharide content, it is unclear if differences in protein content could lead to a potential phenotype.

Lines 339-350 – It is unclear how MVs are quantified. Given the different amounts of LPS, there could be different amounts of MVs produced, skewing possible results. If MVs were not quantified microscopically, results could be questionable due to inconsistent dosage.

Line 650 – 40 ug of MVs is questionable when content of the MVs could greatly differ between WT and mutant bacteria, despite being relatively similar in size.

Reviewer #2: - Growth of the wildtype, ∆lpsB and complemented strains in the conditions of infections should be added to the supplementary as difference in LOS could lead to decrease fitness, impacting the interpretation of experiments in Figure 1.

- Although the pull down gives a qualitative result on the binding of CD14 to LOS, the authors should use an additional more quantitative method to determine the affinity of CD14 to the different LOS

- A lot of details on experimental procedures including bacterial inoculum (CFU), reagents and inhibitors concentration, as well as complete procedures are missing from the material and method, complicating the understanding of some procedures.

- The text should be edited to ensure language as well as scientific clarity.

Reviewer #3: 1. Experimental details with membrane vesicles are not clear, e.g. mouse experiment in Figure 1, Figure 2, Figure 3, .It is not clear how the MV were quantified and normalized for the mouse experiment. The inocula should be normalized for appropriate interpretation. Related, Table S1 shows the lpsB mutant has increased size of MV—how was this measured? If it was by microscopy, please display the images.

2. Some additional controls are needed: In Figure 4e, the GAPDH loading controls must be shown for the 1 h samples and the 6 h samples. For all experiments using inhibitors, controls showing maintenance of viability or secretion of other cytokines/chemokines are needed. E.g. Fig 3b, Fig 3c, Fig 4f, Fig 4i, Fig. 5a.

3. The link between LOS, IFN-B production, and virulence, remains inferred rather than directly interrogated. This should be addressed in the title and results. For Figure 6, the results section over concludes the linking of LOS core-dependent IFN-b signaling to A. baumannii virulence, “These results indicated that the LOS-enhanced IFN-β production was associated with the increased virulence of A. baumannii in the mice.” To test that, IFN-b and/or IFNAR-/- mice could be combined with A. baumannii lpsB mutants: if the model is correct, there should be no difference in virulence between MV from WT v. lpsB in IFNAR-/- or anti-IFN-b-treated mice. Additionally, it is not clear how the fact that IFNAR-/- also blocking IFN-a production would obscure a role for IFNAR in LOS- and CD14-dependent IFN-b production being important. Finally, for Figure 6b, the appropriate control is isotype antibody rather than untreated.

**********

**Part III – Minor Issues: Editorial and Data Presentation Modifications**

Please use this section for editorial suggestions as well as relatively minor modifications of existing data that would enhance clarity.

Reviewer #1: Fig. 1 – Bacteria in figures do not resemble A. baumannii (flagella should not be present).

Fig. 1C – Unclear if statistical testing is between WT vs. mutant, or Complement vs. Mutant.

Line 124 – It is unclear why bacterial burdens would be tested at 4 h when burdens would most likely be different later during infection

Lines 127-129 – Why are IL-1B, IL-6, IL-10, and KC ignored in the results section, when clearly something strange appears to be happening with these levels?

Supplementary Fig. S2b – Infb1 is not highlighted in the volcano plot.

Line 150-152 – This is not a complete sentence.

Fig. 2F – One of the WT heat maps looks completely different from the other 2 replicates. Can the authors explain this?

Lines 213-216 – The inhibitors used are not readily known by some readers, so more explanation may be necessary.

Line 320 – Can the sources, isolation site, isolation year, and the “many antimicrobials” resistant to be noted? This could be of great value to the field.

Lines 340-345. – Centrifugation speed should always be given in x g, as RPM varies by centrifuge.

Line 602 – Does MOI refer to 500 MVs/cell or 500 cells producing MVs/cell

Reviewer #2: - Following experiments in mice, the authors choose to investigate the response of macrophages, and they then alternate between experiments in THP-1 vs J774.A1 cells, it is unclear whether there is a rational for the use of one or the other cell type.

- Some figure sub-panels are not discussed in the text

- The authors do not discuss the inconsistency of other cytokines in between the different experiments. Could the authors speculate on these differences?

- Figure legends should include the description of abbreviations used in the figures

- Kinetics of killing using IP membrane vesicle appears rather inconsistent between experiments, how do the authors choose/normalize the number of vesicles injected?

Did the authors verify that the MV had a similar protein content? What quantity of MV is released by a certain number of bacteria?

- The authors should increase the size of the Figure 1a.

- Figure S1c- according to the literature, the authors should be able to detect the LOS without inner/outer core generated in ∆lpsB mutant – what are the higher molecular weight bands?

- Figure 2f – The color scale is missing.

- Line 147 - The authors should describe the RNA-seq experiment in the text as well as which cells were used.

- Line 152 – It appears that a piece of a sentence is missing

- Line 152-155 - It is unclear in the text why the authors only describe Irf1, Infb1 and Stat2.

- A few references are missing in the results section

Reviewer #3: 1. In the opening sentence of the abstract, virulence typically refers to ability to cause infection by the organism, not molecules such as lipooligosaccharides, so please reword.

2. The manuscript would benefit from additional copyediting throughout.

3. Lines 113-116 appear to be out of place and include figure callouts out of order.

4. Figure 1 A some of the text is too small to read

5. Lines 127-128: Most of the cytokine results are not discussed. It seems odd that the patterns would be so different for some between serum and BALF (e.g. IL-6, KC)—what might these mean for understanding inflammation induced by intact LOS?

6. Why is MIP-2 (murine inflammatory protein 2) shown for Figure 2A with human THP1 macrophages?

7. What are the sequence types of strains shown in Figure 2D?

8. Figure 5: typo-picentannol should be piceatannol

9. Figure 5e: was the difference in pulldown of LOS by CD14 statistically significant?

**********

PLOS authors have the option to publish the peer review history of their article (what does this mean?). If published, this will include your full peer review and any attached files.

If you choose “no”, your identity will remain anonymous but your review may still be made public.

**Do you want your identity to be public for this peer review?**  For information about this choice, including consent withdrawal, please see our Privacy Policy.

Reviewer #1: **Yes:**  Clay D. Jackson-Litteken

Reviewer #2: No

Reviewer #3: No

[NOTE: If reviewer comments were submitted as an attachment file, they will be attached to this email and accessible via the submission site. Please log into your account, locate the manuscript record, and check for the action link "View Attachments". If this link does not appear, there are no attachment files.]

**Figure resubmission:**

While revising your submission, please upload your figure files to the Preflight Analysis and Conversion Engine (PACE) digital diagnostic tool, https://pacev2.apexcovantage.com/. PACE helps ensure that figures meet PLOS requirements. To use PACE, you must first register as a user. Registration is free. Then, login and navigate to the UPLOAD tab, where you will find detailed instructions on how to use the tool. If you encounter any issues or have any questions when using PACE, please email PLOS at figures@plos.org. Please note that Supporting Information files do not need this step. If there are other versions of figure files still present in your submission file inventory at resubmission, please replace them with the PACE-processed versions.

**Reproducibility:**

To enhance the reproducibility of your results, we recommend that authors of applicable studies deposit laboratory protocols in protocols.io, where a protocol can be assigned its own identifier (DOI) such that it can be cited independently in the future. Additionally, PLOS ONE offers an option to publish peer-reviewed clinical study protocols. Read more information on sharing protocols at https://plos.org/protocols?utm_medium=editorial-email&utm_source=authorletters&utm_campaign=protocols

# Author comment: Author response to Decision Letter 1

**DOI:** 10.1371/journal.ppat.1014364.r002

19 Sep 2025

Attachment Submitted filename: Response to Reviewers 20250919.docx

# Peer review: Decision Letter 1

**DOI:** 10.1371/journal.ppat.1014364.r003

**Authors:** Matthew Wolfgang, Mario Feldman, Matthew Wolfgang, Mario Feldman

27 Oct 2025

-->PPATHOGENS-D-25-00850R1

Acinetobacter baumannii lipooligosaccharide core region promotes CD14-dependent TLR4 endocytosis and enhances pathogenicity through interferon-β production

PLOS Pathogens

Dear Dr. Wang,

Thank you for submitting your manuscript to PLOS Pathogens. After careful consideration, we feel that it has merit but does not fully meet PLOS Pathogens's publication criteria as it currently stands. Therefore, we invite you to submit a revised version of the manuscript that addresses the points raised during the review process.

Please submit your revised manuscript within 60 days Dec 26 2025 11:59PM. If you will need more time than this to complete your revisions, please reply to this message or contact the journal office at plospathogens@plos.org. When you're ready to submit your revision, log on to https://www.editorialmanager.com/ppathogens/ and select the 'Submissions Needing Revision' folder to locate your manuscript file.

Please include the following items when submitting your revised manuscript:

* A rebuttal letter that responds to each point raised by the editor and reviewer(s). You should upload this letter as a separate file labeled 'Response to Reviewers'. This file does not need to include responses to any formatting updates and technical items listed in the 'Journal Requirements' section below.

* A marked-up copy of your manuscript that highlights changes made to the original version. You should upload this as a separate file labeled 'Revised Manuscript with Track Changes'.

* An unmarked version of your revised paper without tracked changes. You should upload this as a separate file labeled 'Manuscript'.

If you would like to make changes to your financial disclosure, competing interests statement, or data availability statement, please make these updates within the submission form at the time of resubmission. Guidelines for resubmitting your figure files are available below the reviewer comments at the end of this letter.

We look forward to receiving your revised manuscript.

Kind regards,

Matthew Wolfgang

Section Editor

PLOS Pathogens

Sumita Bhaduri-McIntosh

Editor-in-Chief

PLOS Pathogens

orcid.org/0000-0003-2946-9497

Michael Malim

Editor-in-Chief

PLOS Pathogens

orcid.org/0000-0002-7699-2064

**Additional Editor Comments :**

Two of the original reviewers have re-evaluated this manuscript and remain concerned about several experiments that must be fully addressed in a potential revision. The revised version should experimentally resolve the issue regarding the LOS pattern, as raised by both reviewers. One reviewer noted that “the pulldowns do not appear to show higher affinity for intact LOS to CD14 compared to LBP, as LBP shows ~40 LOS/protein while CD14 shows ~30 LOS/protein.” In the revised version, I expect the authors to provide quantitative measurements of affinity. The issue regarding cytokine responses should also be clarified. If the authors convincingly address these and the additional concerns stated by both reviewers, I would be willing to consider a revised version.

**Journal Requirements:**

1) Regarding Figure 7, thank you for stating " We've redrawn the figure using the open source materials and uploaded the new Fig 7". Please include in the figure legend a link to the source of the images.

Note: If the reviewer comments include a recommendation to cite specific previously published works, please review and evaluate these publications to determine whether they are relevant and should be cited. There is no requirement to cite these works unless the editor has indicated otherwise.

**Reviewers' Comments:**

Reviewer's Responses to Questions

**Part I - Summary**

Please use this section to discuss strengths/weaknesses of study, novelty/significance, general execution and scholarship.

Reviewer #2: (No Response)

Reviewer #3: The authors have addressed some of my previous comments. However, additional information they provide raises new questions on methods and some key experimental comments were not addressed.

**********

**Part II – Major Issues: Key Experiments Required for Acceptance**

Please use this section to detail the key new experiments or modifications of existing experiments that should be absolutely required to validate study conclusions.

Generally, there should be no more than 3 such required experiments or major modifications for a "Major Revision" recommendation. If more than 3 experiments are necessary to validate the study conclusions, then you are encouraged to recommend "Reject".

Reviewer #2: - The data obtained from the mouse experiments (Fig. 1J) appear to be the same as in the original manuscript, except normalized to vesicle number rather than 50 µg of protein. Without repeating the mice experiments, the authors should provide biological replicates demonstrating equivalence between the 50 µg protein and the vesicle number normalization, especially since vesicles from the ΔlpsB strain are larger.

- The authors should repeat and include a LOS silver stain to clearly show the truncated LOS used in this study. Currently, it appears as if no LOS is present in the ΔlpsB strain. For reference, see PMID: 38349180 (Fig 7A), where LOS was extracted from ΔlpsB mutant and displayed a shorter LOS band.

Reviewer #3: 1. Strain construction and Table S3-4:

a. First, ∆lpsB mutants are made in multiple strain backgrounds but only one set of primers is included. Are the sequences where the primers bind actually identical in all three strains? That seems unlikely.

b. Ssecond, the description of the methods would not be likely to generate deletion mutants and there are no methods described or primers listed to verify the deletion mutants. Specifically, using 500 bp homology on either side is low efficiency, when combined with selecting for a double crossover in 1 step, it is extremely unlikely to select correct mutants. Second, there are no primers listed that anneal near the lpsB gene but outside of the construct present in the pGEM-lpsB-apraR that would be required to verify the mutant. Thus, the strains used may be single crossovers integrating pGEM-lpsB-apraR into the chromosome.

2. In Figure S1C, there should be detectable LOS without core sugars in an LOS gel, as previously reported (Hood et al., Infect. Immun., 2013). This comment from reviewer 2 remains a concern.

3. It does not seem appropriate to remove all data for other cytokines as the response to reviewers querying about why the data for other cytokines appearing inconsistent.

4. Line 326-327/Figure 5e: the pulldowns do not appear to show higher affinity for intact LOS to CD14 compared to LBP, as the LBP shows ~40 LOS/protein while CD14 shows ~30 LOS/protein. In addition, as reviewers previously raised, this measure of binding is qualitative and additional methods/analyses should be used to measure affinity quantitatively to support the claims of the manuscript.

**********

**Part III – Minor Issues: Editorial and Data Presentation Modifications**

Please use this section for editorial suggestions as well as relatively minor modifications of existing data that would enhance clarity.

Reviewer #2: - Line 37–38: The current sentence, “Lipooligosaccharides (LOS) with a core region are more pathogenic than those without it; however, the underlying mechanism remains fully elucidated,” is inaccurate, as an LOS molecule itself cannot be described as pathogenic. The sentence should be rephrased in line with line 68–69 which states: “An A. baumannii strain harboring the core region of LOS displayed higher virulence than a strain lacking this core region.” In addition, the phrase “remains fully elucidated” should be corrected to “remains to be fully elucidated.”

- Some figure panels remain too small (e.g., 1B, 1C, 1J), and the labels/writing are difficult to read (e.g., 1D–H).

- Acinetobacter baumannii is not curved, schematics in Fig. 1A, 1I, and S1B should be corrected.

- Fig. 2B: The label “to 1000 ng of LPS” on the y-axis is unclear and should be clarified.

- Fig. 2F: The authors should specify what the scale numbers represent (log₂(FC) or FC).

- Line 181: Please specify the strain/cell type used for RNA-seq.

- Mice were injected with 8.3 × 10¹¹ particles. Given that each bacterium produces ~0.08 vesicles, this corresponds to ~1 × 10¹³ bacteria. Could the authors speculate whether these difference might still be relevant in an infection?

- Fig. 4F: There appear to be issues with the statistical error bars that should be addressed

Reviewer #3: 5. The text in most of the figures is too small

6. The primers to generate complementation vectors are not reported nor any information about pAbYm2 such as antibiotic resistance

7. Table S3 cites reference 48 for the double crossover method, which does not appear to be the correct reference.

8. Antibiotic resistance should be listed/specified for strains used in Table S3 as requested by another reviewer

**********

PLOS authors have the option to publish the peer review history of their article (what does this mean?). If published, this will include your full peer review and any attached files.

If you choose “no”, your identity will remain anonymous but your review may still be made public.

**Do you want your identity to be public for this peer review?**  For information about this choice, including consent withdrawal, please see our Privacy Policy.

Reviewer #2: No

Reviewer #3: No

[NOTE: If reviewer comments were submitted as an attachment file, they will be attached to this email and accessible via the submission site. Please log into your account, locate the manuscript record, and check for the action link "View Attachments". If this link does not appear, there are no attachment files.]

**Figure resubmission:**

-->While revising your submission, we strongly recommend that you use PLOS’s NAAS tool (https://ngplosjournals.pagemajik.ai/artanalysis) to test your figure files. NAAS can convert your figure files to the TIFF file type and meet basic requirements (such as print size, resolution), or provide you with a report on issues that do not meet our requirements and that NAAS cannot fix.

After uploading your figures to PLOS’s NAAS tool - https://ngplosjournals.pagemajik.ai/artanalysis, NAAS will process the files provided and display the results in the "Uploaded Files" section of the page as the processing is complete. If the uploaded figures meet our requirements (or NAAS is able to fix the files to meet our requirements), the figure will be marked as "fixed" above. If NAAS is unable to fix the files, a red "failed" label will appear above. When NAAS has confirmed that the figure files meet our requirements, please download the file via the download option, and include these NAAS processed figure files when submitting your revised manuscript.

**Reproducibility:**

To enhance the reproducibility of your results, we recommend that authors of applicable studies deposit laboratory protocols in protocols.io, where a protocol can be assigned its own identifier (DOI) such that it can be cited independently in the future. Additionally, PLOS ONE offers an option to publish peer-reviewed clinical study protocols. Read more information on sharing protocols at https://plos.org/protocols?utm_medium=editorial-email&utm_source=authorletters&utm_campaign=protocols

# Author comment: Author response to Decision Letter 2

**DOI:** 10.1371/journal.ppat.1014364.r004

11 Mar 2026

Attachment Submitted filename: Response to Reviewers 0311.docx

# Peer review: Decision Letter 2

**DOI:** 10.1371/journal.ppat.1014364.r005

**Authors:** Matthew Wolfgang, Mario Feldman, Matthew Wolfgang, Mario Feldman, Matthew Wolfgang, Mario Feldman

5 Apr 2026

PPATHOGENS-D-25-00850R2

Acinetobacter baumannii lipooligosaccharide core region promotes CD14-dependent TLR4 endocytosis and enhances pathogenicity through interferon-β production

PLOS Pathogens

Dear Dr. Wang,

Thank you for submitting your manuscript to PLOS Pathogens. After careful consideration, we feel that it has merit but does not fully meet PLOS Pathogens's publication criteria as it currently stands. Therefore, we invite you to submit a revised version of the manuscript that addresses the points raised during the review process.

Please submit your revised manuscript by Jun 04 2026 11:59PM. If you will need more time than this to complete your revisions, please reply to this message or contact the journal office at plospathogens@plos.org. When you're ready to submit your revision, log on to https://www.editorialmanager.com/ppathogens/ and select the 'Submissions Needing Revision' folder to locate your manuscript file.

Please include the following items when submitting your revised manuscript:

* A letter that responds to each point raised by the editor and reviewer(s). You should upload this letter as a separate file labeled 'Response to Reviewers'. This file does not need to include responses to any formatting updates and technical items listed in the 'Journal Requirements' section below.

* A marked-up copy of your manuscript that highlights changes made to the original version. You should upload this as a separate file labeled 'Revised Manuscript with Track Changes'.

* An unmarked version of your revised paper without tracked changes. You should upload this as a separate file labeled 'Manuscript'.

If you would like to make changes to your financial disclosure, competing interests statement, or data availability statement, please make these updates within the submission form at the time of resubmission. Guidelines for resubmitting your figure files are available below the reviewer comments at the end of this letter.

We look forward to receiving your revised manuscript.

Kind regards,

Matthew Wolfgang

Section Editor

PLOS Pathogens

Sumita Bhaduri-McIntosh

Editor-in-Chief

PLOS Pathogens

orcid.org/0000-0003-2946-9497

Michael Malim

Editor-in-Chief

PLOS Pathogens

orcid.org/0000-0002-7699-2064

**Additional Editor Comments:**

Overall, the reviewers are pleased with most of the edits and changes in this revised version. However, both reviewers raise concerns about the quality of the SDS-PAGE analysis of LOS (LPS). This issue is not merely cosmetic; the authors need to provide a high-quality silver-stained gel showing the LOS profiles of both the wild-type and mutant strains. One reviewer also suggested including LPS from an additional bacterial species as a reference to aid interpretation. If the authors can improve the SDS-PAGE analysis to the required standard and revise the manuscript in accordance with the reviewers’ comments, the work would be suitable for acceptance.

**Journal Requirements:**

If the reviewer comments include a recommendation to cite specific previously published works, please review and evaluate these publications to determine whether they are relevant and should be cited. There is no requirement to cite these works unless the editor has indicated otherwise.

1) We have noticed that you have cited Tables  S1, and S2 in the manuscript file but there are no corresponding tables in the manuscript.  Please amend your manuscript to include these tables noting that tables should not be uploaded as individual files.

2) We have noticed that you have a list of Supporting Information legends in your manuscript. However, there are no corresponding files uploaded to the submission. Please upload them as separate files with the item type 'Supporting Information'.

**Reviewers' Comments:**

Reviewer's Responses to Questions

**Part I - Summary**

Please use this section to discuss strengths/weaknesses of study, novelty/significance, general execution and scholarship.

Reviewer #2: (No Response)

Reviewer #3: Overall, the additional primer and strain construction information clarifies the methods used and mutant generated. However, the LOS gel remains confusing.

**********

**Part II – Major Issues: Key Experiments Required for Acceptance**

Please use this section to detail the key new experiments or modifications of existing experiments that should be absolutely required to validate study conclusions.

Generally, there should be no more than 3 such required experiments or major modifications for a "Major Revision" recommendation. If more than 3 experiments are necessary to validate the study conclusions, then you are encouraged to recommend "Reject".

Reviewer #2: The authors have addressed most of my comments.

However, the LOS silver stain remains unsatisfactory, both in terms of quality and interpretation (banding pattern).

Reviewer #3: 1. The LOS gel remains confusing. An A. baumannii lpsB mutant has been reported multiple times by multiple groups to generate LOS band slightly lower than the full length LOS (PMID: 23230287; PMID: 32908144; PMID: 38349180). However, these were all with A. baumannii 17978 so it may be different for Ab908. LOS typically migrates more similarly to ~10 kDa molecular weight. The band labeled LOS in Figure S1D at the dye front is not likely LOS, although it is not clear since there were no controls included here for comparison to e.g. rough type E. coli that runs similarly as A. baumannii LOS.

**********

**Part III – Minor Issues: Editorial and Data Presentation Modifications**

Please use this section for editorial suggestions as well as relatively minor modifications of existing data that would enhance clarity.

Reviewer #2: (No Response)

Reviewer #3: 1. Some text in figures remains too small, e.g. the axis labels in Figure 1

2. The lpsB deletion design appears to only delete 35 bp in the middle of the gene. This is fine as a partial deletion/insertion, but should likely be described as a disruption or knockout rather than deletion in the results. The description in the methods in Lines 465-468 is appropriate. However, Line 469-470 should be corrected, as the 500 bp homology arms appear to be within the lpsB gene rather than upstream and downstream of the gene.

3. It seems P8 is not depicted in the primer scheme in Figure S1C.

**********

PLOS authors have the option to publish the peer review history of their article (what does this mean?). If published, this will include your full peer review and any attached files.

If you choose “no”, your identity will remain anonymous but your review may still be made public.

**Do you want your identity to be public for this peer review?**  For information about this choice, including consent withdrawal, please see our Privacy Policy.

Reviewer #2: No

Reviewer #3: No

**Figure resubmission:**

-->While revising your submission, we strongly recommend that you use PLOS’s NAAS tool (https://ngplosjournals.pagemajik.ai/artanalysis) to test your figure files. NAAS can convert your figure files to the TIFF file type and meet basic requirements (such as print size, resolution), or provide you with a report on issues that do not meet our requirements and that NAAS cannot fix.

After uploading your figures to PLOS’s NAAS tool - https://ngplosjournals.pagemajik.ai/artanalysis, NAAS will process the files provided and display the results in the "Uploaded Files" section of the page as the processing is complete. If the uploaded figures meet our requirements (or NAAS is able to fix the files to meet our requirements), the figure will be marked as "fixed" above. If NAAS is unable to fix the files, a red "failed" label will appear above. When NAAS has confirmed that the figure files meet our requirements, please download the file via the download option, and include these NAAS processed figure files when submitting your revised manuscript.

**Reproducibility:**

To enhance the reproducibility of your results, we recommend that authors of applicable studies deposit laboratory protocols in protocols.io, where a protocol can be assigned its own identifier (DOI) such that it can be cited independently in the future. Additionally, PLOS ONE offers an option to publish peer-reviewed clinical study protocols. Read more information on sharing protocols at https://plos.org/protocols?utm_medium=editorial-email&utm_source=authorletters&utm_campaign=protocols

# Author comment: Author response to Decision Letter 3

**DOI:** 10.1371/journal.ppat.1014364.r006

14 May 2026

Attachment Submitted filename: Response to Reviewers 0509.docx

# Editor report: Decision Letter 3

**DOI:** 10.1371/journal.ppat.1014364.r007

**Authors:** Matthew Wolfgang, Mario Feldman, Matthew Wolfgang, Mario Feldman, Matthew Wolfgang, Mario Feldman, Matthew Wolfgang, Mario Feldman

9 Jun 2026

Dear MD Wang,

We are pleased to inform you that your manuscript 'Acinetobacter baumannii lipooligosaccharide core region promotes CD14-dependent TLR4 endocytosis and enhances pathogenicity through interferon-β production' has been provisionally accepted for publication in PLOS Pathogens.

Before your manuscript can be formally accepted you will need to complete some formatting changes, which you will receive in a follow up email. A member of our team will be in touch with a set of requests.

Please note that your manuscript will not be scheduled for publication until you have made the required changes, so a swift response is appreciated.

IMPORTANT: The editorial review process is now complete. PLOS will only permit corrections to spelling, formatting or significant scientific errors from this point onwards. Requests for major changes, or any which affect the scientific understanding of your work, will cause delays to the publication date of your manuscript.

Should you, your institution's press office or the journal office choose to press release your paper, you will automatically be opted out of early publication. We ask that you notify us now if you or your institution is planning to press release the article. All press must be co-ordinated with PLOS.

Thank you again for supporting Open Access publishing; we are looking forward to publishing your work in PLOS Pathogens.

Best regards,

Mario F Feldman

Academic Editor

PLOS Pathogens

Matthew Wolfgang

Section Editor

PLOS Pathogens

Sumita Bhaduri-McIntosh

Editor-in-Chief

PLOS Pathogens

orcid.org/0000-0003-2946-9497

Michael Malim

Editor-in-Chief

PLOS Pathogens

orcid.org/0000-0002-7699-2064

***********************************************************

The new LPS figure is much clearer now

Reviewer Comments (if any, and for reference):

# Editor report: Acceptance letter

**DOI:** 10.1371/journal.ppat.1014364.r008

**Authors:** Matthew Wolfgang, Mario Feldman, Matthew Wolfgang, Mario Feldman, Matthew Wolfgang, Mario Feldman, Matthew Wolfgang, Mario Feldman

Dear MD Wang,

We are delighted to inform you that your manuscript, "Acinetobacter baumannii lipooligosaccharide core region promotes CD14-dependent TLR4 endocytosis and enhances pathogenicity through interferon-β production," has been formally accepted for publication in PLOS Pathogens.

We have now passed your article onto the PLOS Production Department who will complete the rest of the pre-publication process. All authors will receive a confirmation email upon publication.

The corresponding author will soon be receiving a typeset proof for review, to ensure errors have not been introduced during production. Please review the PDF proof of your manuscript carefully, as this is the last chance to correct any scientific or type-setting errors. Please note that major changes, or those which affect the scientific understanding of the work, will likely cause delays to the publication date of your manuscript. Note: Proofs for Front Matter articles (Pearls, Reviews, Opinions, etc...) are generated on a different schedule and may not be made available as quickly.

Soon after your final files are uploaded, the early version of your manuscript, if you opted to have an early version of your article, will be published online. The date of the early version will be your article's publication date. The final article will be published to the same URL, and all versions of the paper will be accessible to readers.

For Research Articles, you will receive an invoice from PLOS for your publication fee after your manuscript has reached the completed accept phase. If you receive an email requesting payment before acceptance or for any other service, this may be a phishing scheme. Learn how to identify phishing emails and protect your accounts at https://explore.plos.org/phishing.

Thank you again for supporting open-access publishing; we are looking forward to publishing your work in PLOS Pathogens.

Best regards,

Sumita Bhaduri-McIntosh

Editor-in-Chief

PLOS Pathogens

orcid.org/0000-0003-2946-9497

Michael Malim

Editor-in-Chief

PLOS Pathogens

orcid.org/0000-0002-7699-2064
